# Probing the Human 4‑Oxo‑l‑proline Reductase-Catalyzed Reaction by Deuterium Kinetic and Equilibrium Isotope Effects

**DOI:** 10.1021/acs.biochem.6c00491

**Published:** 2026-07-16

**Authors:** Ennio Pečaver, Myriam Kabu, Greice M. Zickuhr, David J. Harrison, Rafael G. da Silva

**Affiliations:** † School of Biology, Biomedical Sciences Research Complex, 7486 University of St Andrews, St Andrews KY16 9ST, U.K.; ‡ School of Medicine, University of St Andrews, St Andrews KY16 9TF, U.K.; § NuCana Plc, Edinburgh EH12 9DT, U.K.

## Abstract

Human 4-oxo-l-proline reductase (*Hs*BDH2), a short-chain dehydrogenase/reductase (SDR) superfamily member possessing a canonical N–S–Y–K catalytic tetrad, catalyzes the formation of the endogenous anticancer compound *cis*-4-hydroxy-l-proline via the NADH-dependent reduction of 4-oxo-l-proline. Except for hydride-transfer stereochemistry, information on the *Hs*BDH2 chemical step remains elusive. Here, deuterium isotope effects are employed to gather information on the hydride-transfer step of the reaction. Primary and α-secondary equilibrium isotope effects were inverse and normal, respectively, consistent with the 4*S*-[^2^H] accumulating on *cis*-4-hydroxy-l-proline and the 4*R*-[^2^H] on NADH. Primary kinetic isotope effects, with 4*S*-[4-^2^H]­NADH, on *k*_cat_ (^D^ *k*_cat_) and *k*_cat_/*K*_M_ (^D^(*k*_cat_/*K*_M_^4OLP^)) were small, indicating that hydride transfer is fast relative to other catalytic steps. Internal-competition equilibrium binding isotope effects were inverse with 4*S*-[4-^2^H]­NADH and normal with 4*R*-[4-^2^H]­NADH, suggesting that *Hs*BDH2 tight binding distorts the pro-*S* hydrogen to a more constrained bonding environment, while the opposite happens to the pro-*R* hydrogen. Both levulinate and pyruvate were low-affinity, slow-reaction substrates; while a ^D^(*k*_cat_/*K*_M_) of 2.6 and a ^D^ *k*_cat_ of 2.5 were determined with the former, modest values were obtained with the latter. Using NADPH as a coenzyme resulted in a similar *k*_cat_, but drastically increased *K*_M_. Accordingly, 4*S*-[4-^2^H]­NADPH increased ^D^(*k*_cat_/*K*_M_^NADPH^) to 1.5, while ^D^ *k*_cat_ remained small. The N105A substitution, proposed to affect coenzyme binding in SDRs, increased *K*_M_ for NADH and doubled *k*_cat_. The ^D^(*k*_cat_/*K*_M_) increased to 2.5 and ^D^ *k*_cat_ to 2.0. The ^D_2_O^ *k*_cat_ with 4*S*-[4-^2^H]­NADH decreased from its value with NADH, suggesting stepwise hydride-transfer and proton-transfer steps. These results expand the role of N105 in *Hs*BDH2 and possibly other SDRs.

## Graphical abstract

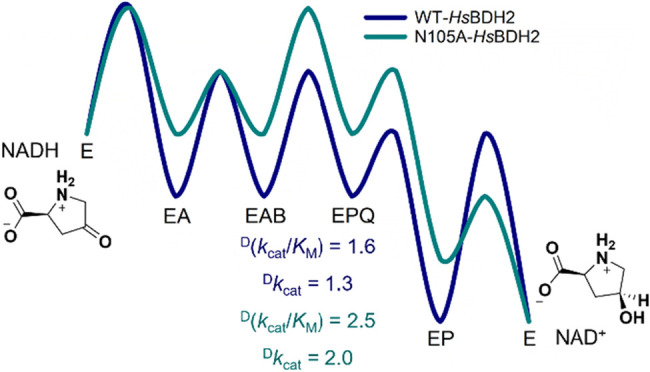

## Introduction

Human 4-oxo-l-proline reductase (*Hs*BDH2) (EC 1.1.1.104) is a cytosolic enzyme that catalyzes the NADH-dependent reduction of 4-oxo-l-proline, generating NAD^+^ and *cis*-4-hydroxy-l-proline^1^ (Scheme 1), an endogenous compound with known anticancer properties.^2^ Before the physiological substrate of *Hs*BDH2 was discovered, the crystal structure of the enzyme had been solved in complex with NAD^+^,^3^ and its primary and tertiary structures place it within the short-chain dehydrogenase/reductase (SDR) superfamily, one of the largest protein families with at least 80 encoding genes identified or predicted in humans.^4^

**Scheme 1:**
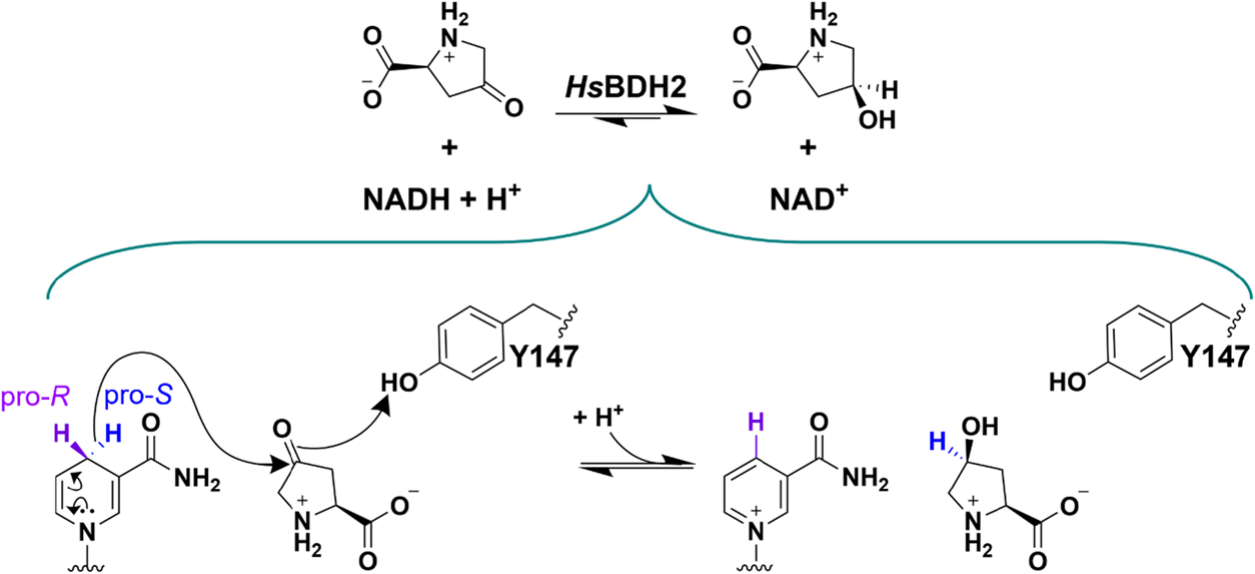
*Hs*BDH2-Catalyzed Reversible Reduction of 4-Oxo-l-proline, with the Pro-*S* Hydrogen in Blue and the Pro-*R* in Purple

SDRs feature a Rossman fold for NAD­(P)H binding and typically a conserved N–S–Y–K catalytic tetrad.^5,6^ The tyrosine residue is often the general acid in reduction reactions, aided by the lysine residue as part of a proton-relay system involving water molecules and the 2′-OH group of the coenzyme. The serine residue helps position the substrate for catalysis.^7^ The role of the asparagine is not well-established, but it has been proposed to be important for coenzyme binding.^8,9^ *Hs*BDH2 has a marked preference for NADH over NADPH, and the catalytic tetrad consists of residues N105, S133, Y147, and K151.^3^

Our previous work with deuterated NADH isotopologues established that *Hs*BDH2 transfers the pro-*S* hydrogen from NADH to 4-oxo-l-proline (Scheme 1). We also determined that although the reaction is reversible, the equilibrium is strongly displaced toward NAD^+^ and *cis*-4-hydroxy-l-proline formation (*K*_eq_ of ∼1.4 × 10^11^ M^–1^). We discovered that *Hs*BDH2 binds tightly to NADH (*K*_D_ of 0.48 μM) and is copurified with the coenzyme. Pre-steady-state kinetics suggested that *k*_cat_ is not limited by chemistry, but by NAD^+^ release in a steady-state ordered kinetic mechanism (Scheme 2). Interestingly, the single-turnover rate constant is only twice as high as *k*_cat_, suggesting that the interconversion between *Hs*BDH2:NADH:4OLP and *Hs*BDH2:NAD^+^:C4HLP ternary complexes might contribute to the overall reaction rate.^10^ However, information on the hydride-transfer step itself and its contribution to the reaction rate is still missing.

**Scheme 2:**
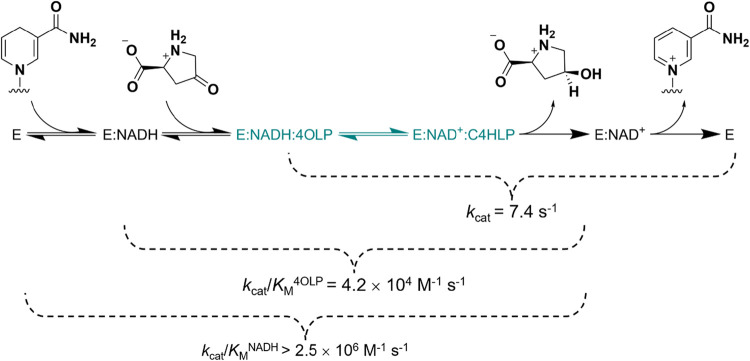
*Hs*BDH2 Proposed Kinetic Sequence and Experimental Steady-State Rate Constants at 25 °C and pH 7.0^a^

a The hydride-transfer step is in teal.

In the present work, we aim to gain insight into the chemical step of the reaction and elucidate the role of the conserved N105 in catalysis. We employ a combination of equilibrium and kinetic isotope effects of deuterium, including solvent and multiple isotope effects, site-directed mutagenesis, and pre-steady-state kinetics. Our results shed light on hydride transfer by *Hs*BDH2 and may be relevant for the role of the conserved catalytic tetrad residue N105 in other SDRs.

## Materials and Methods

### Materials

All chemicals were used without further purification or modification. Ampicillin and dithiothreitol (DTT) were purchased from Formedium. *E. coli* DH5α (high efficiency) and Q5 DNA polymerase were purchased from New England Biolabs. NADH, NADPH, NADP^+^, NAD^+^, ethylenediaminetetraacetic acid-free complete protease inhibitor cocktail, 2-(N-morpholino)­ethanesulfonic acid (MES), 4-oxo-l-proline, acetoacetate, lysozyme, 2-(cyclohexylamino)­ethanesulfonic acid (CHES), d-glucose, deuterium-labeled d-glucose (1-d_1_, 98% deuterium), formic acid, deuterium-labeled formic acid (formic-d acid, 98% deuterium), *Pseudomonas sp*. glucose dehydrogenase, *Candida boidinii* formate dehydrogenase, and deuterium oxide (D_2_O) (99.9% deuterium) were purchased from Merck. Agarose, 4-(2-Hydroxyethyl)­piperazine-1-ethanesulfonic acid (HEPES), *cis*-4-hydroxy-l-proline, dNTPs, and NaCl were purchased from Thermo Fisher Scientific. WT-*Hs*BDH2 was produced as previously described.^10^ N105A-*Hs*BDH2 was produced by the same protocol as WT-*Hs*BDH2, and its exact mass was determined by LC-ESI-MS analysis at the University of St Andrews Proteomics and Mass Spectrometry Facility. Compounds tested as alternative substrates for *Hs*BDH2, replacing 4-oxo-l-proline, were purchased from Merck and are listed in Table S1.

### Synthesis of NAD­(P)­H

For isotope effect measurements, NADH, NADPH, and their respective isotope-labeled versions were synthesized and purified in-house. NADH and 4*S*-[4-^2^H]­NADH (pro-*S* NADD) were prepared as previously described.^11^ 4*R*-[4-^2^H]­NADH (pro-*R* NADD) was produced as previously reported.^10^ NADPH and 4*S*-[4-^2^H]­NADPH (pro-*S* NADPH) were prepared by the same protocol as NADH and 4*S*-[4-^2^H]­NADH, except that NADP^+^ replaced NAD^+^. All isotope effect measurements involving deuterated and non-deuterated NAD­(P)H employed a coenzyme prepared in-house.

### Site-Directed Mutagenesis

The N105A mutation was introduced by the overlapping primers method,^12^ using the WT-*Hs*BDH2 expression construct as the template. The forward and reverse primers were 5′-GAATTTAGCCGTACGCAGCATGTACCTTATGATCAAAGCC-3′ and 5′-CTTTTTCTGACCCTAAAGTCGTACTTAAATCGGCATGCGTC-3′, respectively. Correct insertion was confirmed by DNA sequencing (Eurofins Genomics).

### *Hs*BDH2 Activity Assay

Unless stated otherwise, *Hs*BDH2 enzymatic activity was assayed at 25 °C in 500 μL in 100 mM HEPES (pH 7.0), 12.5 mM NaCl, and 1 mM DTT, following the decrease in absorbance at 340 nm owing to NADH oxidation (ε_340_ = 6220 M^–1^ cm^–1^) in a 1 cm path length quartz cuvette, as previously described.^10^ To screen WT-*Hs*BDH2 activity against compounds listed in Table S1 as alternative substrates to 4-oxo-l-proline, 1 mM compound was used, pH-adjusted to 7.0, 24 μM NADH, and either 10 or 400 nM enzyme. In all cases, controls lacked the enzyme. For the 4-oxo-l-proline stock solution, the pH of the solution (in water) was adjusted to ∼5, and for all other substrates, the pH of the stock solution was brought to ∼7, to ensure that even at high substrate concentrations, the pH of the reaction in the cuvette would not be altered, which was confirmed by measuring the pH of the reaction after rate measurement.

### Primary Deuterium Kinetic Isotope Effects

All kinetic isotope effects were determined by the direct comparison method. Primary deuterium kinetic isotope effects were measured with 20 nM WT-*Hs*BDH2, a series of fixed concentrations (6–24 μM) of either NADH or pro-*S* NADD, and varying 4-oxo-l-proline concentrations (0–2.0 mM). Alternatively, primary deuterium kinetic isotope effects were measured with 800 nM WT-*Hs*BDH2, 24 μM either NADH or pro-*S* NADD, and varying concentrations of either pyruvate (0–16 mM) or levulinate (0–131 mM). Three independent measurements were carried out. Primary deuterium kinetic isotope effects on *k*_cat_ (^D^ *k*_cat_) and *k*_cat_/*K*_M_ (^D^(*k*_cat_/*K*_M_)) were determined.

### Dependence of Deuterium Isotope Effects on pH

The ^D^ *k*_cat_ and ^D^(*k*_cat_/*K*_M_^4OLP^) were further measured across a pH range of 6.0–9.0 at 1 pH unit increments by measuring initial rates in composite buffer (100 mM MES, 100 mM HEPES, 100 mM CHES, 12.5 mM NaCl, and 1 mM DTT).^10^ Reactions were carried out in triplicate determinations using 20 nM WT-*Hs*BDH2, 24 μM either NADH or pro-*S* NADD, and 0–7.5 mM 4-oxo-l-proline. Three independent measurements were carried out. Solvent deuterium kinetic isotope effects on *k*_cat_ (^D_2_O^ *k*_cat_) and *k*_cat_/*K*_M_^4OLP^ (^D_2_O^(*k*_cat_/*K*_M_^4OLP^)) were carried out in either H_2_O or D_2_O as the solvent used in buffer preparation and solubilization of all reagents except the enzyme (final D_2_O proportion (v/v): 99.6%). Initial velocities were measured across a pH or pD range of 6.5–9.5 at 0.5 pH­(D) unit increments, where pD = pH meter reading +0.4. Reactions were carried out in the composite buffer system using 10 nM WT-*Hs*BDH2, 24 μM NADH, and 0–16.4 mM 4-oxo-l-proline. Two independent measurements were carried out. The pH of the reaction was confirmed by measuring it after rate determination.

### Deuterium Equilibrium Isotope Effects

Primary and α-secondary equilibrium isotope effects were measured at 25 °C in a direct comparison method initially described by Cook et al.^13^ and modified by Czekster et al.^14^ All 500-μL reactions were monitored spectrophotometrically at 340 nm in 1 cm optical path length quartz cuvettes (Hellma) using a Shimadzu UV-2600 spectrophotometer outfitted with a CPS unit for temperature control. At least six independent reactions were measured for each isotopologue in each experiment. These measurements were recorded across multiple days.

For the primary deuterium equilibrium isotope effect (^D^ *K*_eq_), solutions containing assay buffer, 50 μM of either NADH or pro-*S* NADD, and 500 μM 4-oxo-l-proline were prepared. These were incubated at 25 °C in the spectrophotometer while recording absorbance at 340 nm for 3 min to establish a signal baseline. Following the incubation, 1 μL of *Hs*BDH2 was added to obtain a final concentration of 41.6 nM while minimizing the absorbance baseline perturbation. The reaction absorbance was monitored for 20 min. Absorbance levels at equilibrium were obtained by averaging the absorbance values at the end of the reaction time course (800 s). The difference in absorbance between the 3 min incubation baseline average and the equilibrium average was converted to the total amount of NADH or pro-*S* NADD depleted (ε = 6220 M^–1^ cm^–1^). The α-secondary deuterium equilibrium isotope effect (^α‑D^ *K*_eq_) was determined under the same conditions and by the same procedure, except that pro-*R* NADD substituted for pro-*S* NADD and the reaction time course was 600 s. The following assumptions were made: the concentration of NAD^+^ formed in the reaction is equal to the amount of either NADH or NADD consumed; the concentration of *cis*-4-hydroxy-l-proline formed is equal to the concentration of NAD^+^ formed; and the remaining concentration of 4-oxo-l-proline is equal to the starting concentration minus the equivalent of formed *cis*-4-hydroxy-l-proline. Negative controls lacking the enzyme were incubated for the same duration as the reactions.

### Binding Isotope Effects

The primary deuterium binding isotope effect (^D^ *K*_A_) is defined as the ratio of the equilibrium association constant (*K*_A_) with pro-*S* NADH to that with pro-*S* NADD. The α-secondary deuterium binding isotope effect (^α‑D^ *K*_A_) is the ratio of the *K*_A_ pro-*R* NADH to that with pro-*R* NADD.^15^ The ^D^ *K*_A_ and ^α‑D^ *K*_A_ were measured by the internal-competition method using Rapid Equilibrium Dialysis (RED) plate and inserts (Thermo Fisher)^16^ and analyzing the fractions by high-resolution liquid chromatography-electrospray ionization-mass spectrometry (LC-ESI-MS). Deconvolution of peaks was performed based on the method developed by Linscott et al.^17^

LC-ESI-MS analyses of NADH, pro-*S*, and pro-*R* NADD were performed as previously reported.^10^ Analysis of individual standards yielded three peaks corresponding to NADH (*m*/*z* 664.1227, 665.1257, and 666.1293) and to either pro-*S* or pro-*R* NADD (*m*/*z* 665.1257, 666.1293, and 667.1357). Additional peaks were also present at higher *m*/*z* for NADH (*m*/*z* 686.1046, 687.1076, and 688.1112) and either pro-*S*- or pro-*R*-NADD (*m*/*z* 687.1076, 688.1112, and 689.1176), corresponding to the coenzyme-Na^+^ adducts. Triplets of peaks stem from the natural isotope abundances of NADH. Areas of corresponding peaks from non-adduct and Na^+^-adduct peaks (*m*/*z* 664.1227 and 686.1046, *m*/*z* 665.1257 and 687.1076, and *m*/*z* 666.1293 and 688.1112) were summed and used to establish the ratio of peak areas among the different natural isotope abundance species (1:0.267:0.059 in the experiment to measure the pro-*S* deuterium binding isotope effect, and 1:0.255:0.061 in the experiment to measure the pro-*R* deuterium binding isotope effect). These ratios were used to deconvolute the LC-ESI-MS spectra from the binding isotope effect experiments described below.

To prepare samples for isotope effects measurement, each RED well insert on the RED plate contained an “in” dialysis bag component (50 μL), and an “out” assay buffer (100 mM HEPES pH 7.0, 12.5 mM NaCl, and 1 mM DTT) component (300 μL) as per the manufacturer’s instructions. The “in” component contained assay buffer, 20 μM *Hs*BDH2, 150 μM NADH, and 150 μM either pro-*S* or pro-*R* NADD. The “out” component contained assay buffer. The RED plate was covered with aluminum foil and placed in a 25 °C incubator, shaking gently for 4 h to equilibrate across the RED insert dialysis membrane. After equilibration, all “in” and “out” samples were retrieved by pipetting. The enzyme was removed from the “in” samples by treating the solution with 100 μL of methanol, vortexing, and centrifuging at 11,500*g*, after which the methanol was removed using a Thermo Savant SPD1010 vacuum centrifugation system (Thermo Fisher). Each sample was analyzed by LC-ESI-MS as previously described.^10^ Negative controls were prepared and analyzed in an identical manner, except no enzyme was added to the “in” component.

High-resolution LC-ESI-MS analysis resulted in peaks corresponding to NADH and either pro-*S* or pro-*R* NADD with the corresponding *m*/*z* as described above, including the Na^+^ adducts. The mixture of isotopologues resulted in packets of four non-adduct peaks [M–H]^−^ and four Na^+^ adduct peaks [M–2H+Na]^−^ with contributions from both NADH and either pro-*S* or pro-*R* NADD (*m*/*z* 665.1257 and 666.1293 in the non-adduct peaks and *m*/*z* 687.1076 and 688.1112 in the Na^+^ adduct peaks). After summing the areas of corresponding non-adduct and Na^+^ adduct peaks, the ratios from the NADH standards described above were used to deconvolute the peaks with contributions from different isotopologues, i.e., to calculate the contribution of NADH and of either pro-*S* or pro-*R* NADD to each peak. The first peak has contributions exclusively from NADH. The second and third peaks have contributions from both NADH and NADD; the ratio of the first and second peaks in the standard is then used to calculate the contributions from NADH, with the remaining corresponding to NADD. The same process is repeated for the third peak by using the ratios of the first and third peaks in the standard to determine the contributions from NADH and NADD. Finally, the fourth peak has contributions exclusively from NADD. The sum of each of the peak areas was then used to derive the total peak area for the isotopologue.

A minimum of seven injections were analyzed by LC-ESI-MS for the NADH standards used to calculate peak area ratios. For the binding isotope effects with a mixture of NADH and pro-*R* NADD, 13 independent reactions were prepared, as well as three negative controls, and ten injections of each reaction were analyzed by LC-ESI-MS. For the binding isotope effects with a mixture of NADH and pro-*S* NADD, three independent reactions and three negative controls were prepared, and five injections of each reaction were analyzed by LC-ESI-MS.

Errors were calculated and propagated as follows: for the NADH standards used to determine the peak ratios, the uncertainty was calculated using the standard error of the mean (SEM) peak area across all injections, including those corresponding to NADH–Na^+^ adducts. The mean peak area and SEM were calculated across the five injections for each reaction, and this error was propagated with the SEM of the standards when deconvoluting the peaks to calculate the contributions from NADH and NADD. For each reaction, a binding isotope effect and propagated SEM were calculated by applying the required values to the appropriate equation (see below). The final binding isotope effect is reported as mean ± propagated SEM across the independent reactions. The raw data and the calculations leading to ^D^ *K*_A_ and ^α‑D^ *K*_A_ have been deposited in the University of St Andrews Pure research repository and can be accessed at 10.17630/821f4347-65de-46d0-9459-e01b1261f9b0.

### Steady-State Kinetics with NADPH as a Coenzyme

All 500-μL reactions in assay buffer were monitored spectrophotometrically at 370 nm for 1.5 min under initial rate conditions in 0.5 cm optical path length quartz cuvettes (Hellma) at 25 °C using a Shimadzu UV-2600 spectrophotometer outfitted with a CPS unit for temperature control. Activity was monitored via the decrease in absorbance corresponding to NADPH oxidation (ε_370_ = 2320 M^–1^ cm^–1^). Reactions contained 20 nM WT-*Hs*BDH2. Substrate saturation curves were determined by varying either 4-oxo-l-proline (0–4.1 mM) at 1.3 mM NADPH or NADPH (0–2.5 mM) at 2 mM 4-oxo-l-proline. Two independent rate measurements were carried out. Controls lacked the enzyme.

The ^D^ *k*_cat_ and ^D^(*k*_cat_/*K*_M_) were measured at 25 °C by varying either NADPH (0–1.6 mM) at 2.0 mM 4-oxo-l-proline or pro-*S* NADPD (0–1.5 mM) at 4.1 mM 4-oxo-l-proline. Alternatively, either NADPH or pro-*S* NADPD was held at 1.6 mM, and 4-oxo-l-proline was varied (0–4.1 mM). Three independent rate measurements were carried out. Controls lacked the enzyme.

### Steady-State Kinetics with N105A-*Hs*BDH2

All 500-μL reactions in assay buffer were monitored spectrophotometrically at 370 nm for 2 min under initial rate conditions in 0.5 cm optical path length quartz cuvettes (Hellma) at 25 °C using a Shimadzu UV-2600 spectrophotometer outfitted with a CPS unit for temperature control. Activity was monitored via the decrease in absorbance corresponding to NADH oxidation (ε_370_ = 2320 M^–1^ cm^–1^). Reactions contained 20 nM N105A-*Hs*BDH2. Substrate saturation curves were determined by varying either 4-oxo-l-proline (0–6.1 mM) at 1.536 mM NADH or NADH (0–1.5 mM) at 6.1 mM 4-oxo-l-proline. Two independent rate measurements were carried out. Controls lacked the enzyme.

The ^D^ *k*_cat_ and ^D^(*k*_cat_/*K*_M_) with N105A-*Hs*BDH2 were measured at 25 °C by either varying 4-oxo-l-proline (0–12.3 mM) at 768 μM, either NADH or pro-*S* NADD, or varying either NADH or pro-*S* NADD (0–768 μM) at 12.3 mM 4-oxo-l-proline, with 138 nM N105A-*Hs*BDH2. Three independent rate measurements were carried out. Controls lacked the enzyme.

### Pre-Steady-State Kinetics with N105A-*Hs*BDH2

N105A-*Hs*BDH2 rapid kinetics under multiple-turnover conditions were performed at 25 °C in an Applied Photophysics SX-20 stopped-flow spectrophotometer outfitted with a 5-μL mixing cell (0.5 cm path length, 0.9 ms dead time) and a circulating water bath for temperature control. Reactions were triggered by rapid mixing of 55 μL from each syringe, and the depletion of NADH measured via absorbance decreased at 370 nm. Each syringe contained assay buffer. One syringe contained 1.6 mM NADH and 30 μM N105A-*Hs*BDH2, and the other contained 12.3 mM 4-oxo-l-proline. A total of 9 traces were collected, each containing 5,000 data points over 0.5 s in a linear time scale. Controls lacked the enzyme.

### N105A-*Hs*BDH2 pH- and pD-Rate Profiles

Initial velocities were measured across a pH or pD range of 6.5–9.0 at 0.5 pL unit increments, where pD = pH meter reading +0.4, at 25 °C in the presence of 138 nM N105A-*Hs*BDH2, varying concentrations of 4-oxo-l-proline (0–24.6 mM 4-oxo-l-proline, pH 6.5–7.5 and pH 8.5–9.0; 0–12.3 mM 4-oxo-l-proline, pH 8.0) and fixed, saturating concentrations of NADH (1.6 mM, pH 6.5 and 7.5; 0.8 mM, pH 7.0; 1.2 mM NADH, pH 8.0–9.0) in the same composite buffer used for the pH- and pD-rate profiles with WT-*Hs*BDH2. Two independent measurements were carried out. The pH of the reaction was confirmed by measuring it after rate determination. Prior to the experiment, to test enzyme stability at the extremes of the pH range, enzymes were diluted in buffer at either pH 6.5 or 9.0 prior to activity assay at pH 7.0.

### N105A-*Hs*BDH2 Primary Deuterium Kinetic Isotope Effects at pH 7.5

The ^D^ *k*_cat_ and ^D^(*k*_cat_/*K*_M_) with N105A-*Hs*BDH2 were measured at 25 °C in 100 mM HEPES (pH 7.5), 12.5 mM NaCl, and 1 mM DTT by either varying 4-oxo-l-proline (0–12.3 mM) at 0.8 mM either NADH or pro-*S* NADD, or varying either NADH or pro-*S* NADD (0–0.8 mM) at 12.3 mM 4-oxo-l-proline, with 138 nM N105A-*Hs*BDH2. Three independent rate measurements were carried out. Controls lacked the enzyme.

### N105A-*Hs*BDH2 Multiple Deuterium Kinetic Isotope Effects at pH 7.5

The ^D_2_O^ *k*_cat_ with N105A-*Hs*BDH2 was measured at 25 °C in 100 mM HEPES (pL 7.5), 12.5 mM NaCl, and 1 mM DTT in either H_2_O or 99% (v/v) D_2_O by varying 4-oxo-l-proline (0–12.3 mM) at a fixed, saturating concentration of either NADH or pro-S NADD and 138 nM N105A-*Hs*BDH2. Three independent rate measurements were carried out. Controls lacked the enzyme.

### Substrate Saturation Curves in the Presence of Glycerol

Initial rates were measured with 10 nM WT-*Hs*BDH2 in 100 mM tricine (pH 8.0), 12.5 mM NaCl, 1 mM DTT, 24 μM NADH, and varying concentrations of 4-oxo-l-proline (0–2.0 mM) at 25 °C in the presence or absence of 9% (v/v) glycerol. Alternatively, initial rates were measured with 138 nM N105A-*Hs*BDH2 in 100 mM HEPES (pH 7.5), 12.5 mM NaCl, and 1 mM DTT at either 0–12.3 mM 4-oxo-l-proline and 1.5 mM NADH or 0–1.5 mM NADH and 12.3 mM 4-oxo-l-proline at 25 °C in the presence or absence of 9% (v/v) glycerol.

### Data Analysis

Kinetics and equilibrium data were analyzed by the non-linear regression function of SigmaPlot 13.0 (SPSS). The apparent *K*_eq_ (*K*_eq_^app^) was calculated with eq 1. The ^D^ *K*_eq_ and ^α‑D^ *K*_eq_ were calculated by dividing the *K*_eq_^app^ with the light isotope by the *K*_eq_^app^ with the heavy isotope.^13^ Substrate saturation curves at a fixed concentration of the co-substrate were fitted to eq 2. Kinetic isotope effects on both *k*_cat_ and *k*_cat_/*K*_M_ were fitted to eq 3 and, on *k*_cat_ only, to eq 4, except those calculated for the pH-dependence of the isotope effect, which were calculated by dividing the rate constant with the light isotope by the corresponding rate constant with the heavy isotope. The pH-rate profiles were fitted to eq 5, and the pH-dependence of the kinetic isotope effects were fitted to eq 6.^14,18^ The ^D^ *K*_A_ and ^α‑D^ *K*_A_ were calculated with eq 7, analogous to that derived by Murkin et al.^15^ In eqs 1–7, *v* is the initial rate, *k*_cat_ is the steady-state catalytic rate constant, *K*_M_ is the Michaelis constant, *E*_T_ is total enzyme concentration, *S* is the concentration of the substrate, [NAD^+^] and [NADH] are the concentrations of the oxidized and reduced coenzyme, respectively, [4OLP] and [C4HLP] are the concentrations of 4-oxo-l-proline and *cis*-4-hydroxy-l-proline, respectively, *F*_i_ is the fraction of the deuterium label (0.98, assumed based on the 98% deuterium content in d-glucose 1-d_1_), *E*_*k*_cat_/*K*_M__ and *E*_*k*_cat__ are the ^D^(*k*_cat_/*K*_M_) minus 1 and the ^D^ *k*_cat_ minus 1, respectively, *C* is the pH-independent value of *k*_cat_, *H* is the proton concentration, *K*_a1_ and *K*_a2_ are apparent acid dissociation constants, p*K*_a_ is −log­(*K*_a_), *Y*_L_ and *Y*_H_ are the kinetic isotope effect plateaus at low pH and high pH, respectively, NADL_in_ and NADL_out_ (where L stands for either H or D) are the sum of NADL peaks in the “in” and “out” components of RED, respectively, and the subscript X stands for either D or α-D.

1 $$K_{\mathrm{eq}}^{\mathrm{app}}=\frac{{[\mathrm{NAD}}^{+}]\left[C4\mathrm{HLP}\right]}{\left[\mathrm{NADL}\right]\left[4\mathrm{OLP}\right]}$$

2 $$\frac{v}{E_{T}}=\frac{k_{\mathrm{cat}}S}{K_{M}+S}$$

3 $$\frac{v}{E_{T}}=\frac{k_{\mathrm{cat}}S}{K_{M}\left(1+F_{i}E_{k_{\mathrm{cat}}/K_{M}}\right)+S\left(1+F_{i}E_{k_{\mathrm{cat}}}\right)}$$

4 $$\frac{v}{E_{T}}=\frac{k_{\mathrm{cat}}S}{K_{M}+S\left(1+F_{i}E_{k_{\mathrm{cat}}}\right)}$$

5 $$\log k_{\mathrm{cat}}=\log\left(\frac{C}{1+\left(\frac{H}{K_{a1}}\right)+\left(\frac{K_{a2}}{H}\right)}\right)$$

6 kcatDor(kcat/KM)D=YL+YH(10−pKa10−pH)1+(10−pKa10−pH)

7 KAX=(NADHinNADHout−1)(NADDinNADDout−1)

## Results and Discussion

### Deuterium Equilibrium Isotope Effects for Reduction of 4-Oxo-l-proline by NADH

Equilibrium isotope effects reflect the propensity of the heavy isotope to accumulate in a molecule (either substrate or product) where the bond is “stiffer”, or a more constrained bonding environment, when the reaction reaches equilibrium. The stiffness of the C–H bond increases with the number of heavy-atom substituents on an sp^3^ carbon, and deuterium will become enriched in the more substituted of the two sp^3^ carbons between which it is being transferred. When the hydrogen in question is not being transferred, but the carbon to which it is bonded undergoes a change in hybridization from sp^3^ to sp^2^, the protium will accumulate on the sp^2^ carbon due to increased out-of-plane bending vibration.^13,19,20^ In the case of the *Hs*BDH2-catalyzed reaction, this rationale predicts that deuterium will be enriched in the *cis*-4-hydroxy-l-proline instead of NADH when pro-*S* NADD is used because of the oxygen substituent on the C4 of the amino acid, and in NADH instead of NAD^+^ when pro-*R* NADD is used, given the sp^3^ hybridization on the C4 of the reduced nicotinamide ring. This is what we qualitatively obtained when we determined ^D^ *K*_eq_ and ^α‑D^ *K*_eq_ (Figure 1): an inverse ^D^ *K*_eq_ of 0.76 ± 0.05, consistent with deuterium enrichment in *cis*-4-hydroxy-l-proline, and a normal ^α‑D^ *K*_eq_ of 1.12 ± 0.02, indicating deuterium accumulation on the sp3 C4 of NADH as opposed to the sp^2^ C4 of NAD^+^. This is in contrast to the normal ^D^ *K*_eq_ of 1.14 reported for the NADPH-dependent reduction of dihydrofolate catalyzed by dihydrofolate reductase despite a nitrogen substituent on the hydride-acceptor carbon.^14^

**Figure 1:**
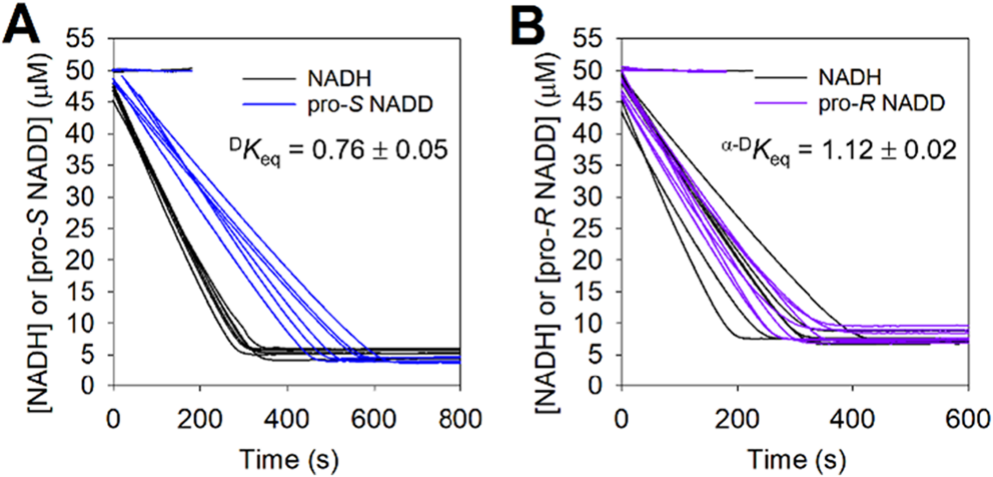
Equilibrium deuterium isotope effects. (A) *Hs*BDH2-catalyzed reaction with either NADH or pro-*S* NADD to determine ^D^ *K*_eq_. (B) *Hs*BDH2-catalyzed reaction with either NADH or pro-*R* NADD to determine ^α‑D^ *K*_eq_. The flat traces at ∼50 μM coenzyme are controls lacking the enzyme, which served as a baseline for concentration determination. Both ^D^ *K*_eq_ and ^α‑D^ *K*_eq_ values are mean ± SEM of at least six independent reactions. All measurements (lines) are shown.

### Primary Deuterium Kinetic Isotope Effects on WT-*Hs*BDH2-Catalyzed 4-Oxo-l-proline Reduction

To gauge the contribution of the hydride-transfer step to the WT-*Hs*BDH2-catalyzed reaction, we measured ^D^(*k*_cat_/*K*_M_^4OLP^) and ^D^ *k*_cat_ (Figure 2A–C), with values reported in Table 1. The ^D^(*k*_cat_/*K*_M_^4OLP^) and ^D^ *k*_cat_ remained small at different levels of NADL, suggesting that hydride transfer contributes only modestly to the reaction rate at low and high 4-oxo-l-proline levels. When varying the coenzyme, we could only determine ^D^ *k*_cat_ accurately (Figure 2D) due to the limitations of our assay at low coenzyme concentrations and the tight binding of NADH, and apparently pro-*S* NADD, to WT-*Hs*BDH2.^10^ Analyzing the data in Figure 2B in the double-reciprocal form (Figure S1A) produces the same ^D^ *k*_cat_ (from the ratio of *y*-axis intercepts) and suggests a ^D^(*k*_cat_/*K*_M_^NADH^) trending to ∼1 from the ratio of slopes.

**Figure 2:**
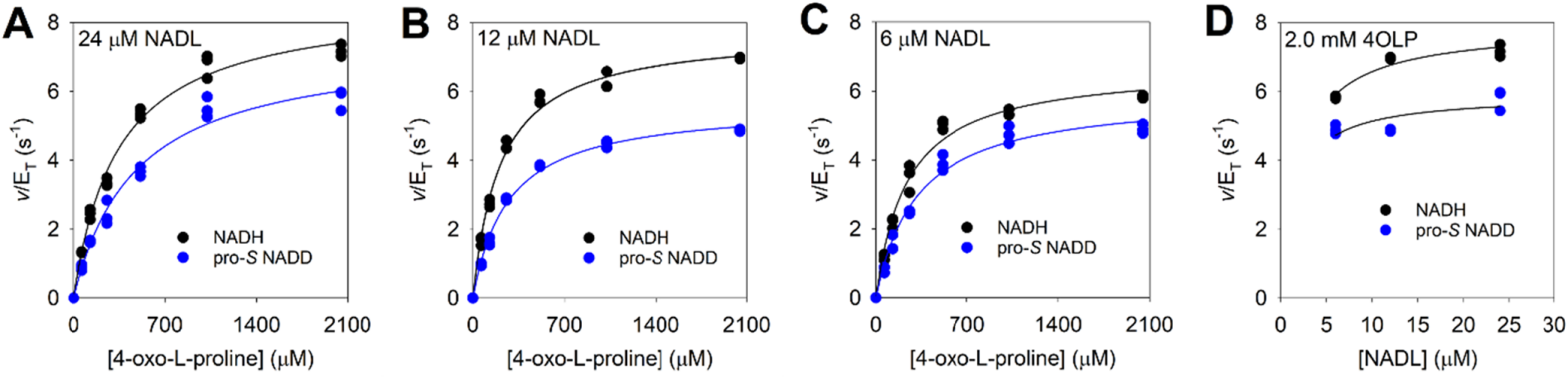
Primary deuterium kinetic isotope effects on WT-*Hs*BDDH2-catalyzed 4-oxo-l-proline reduction by NADH. (A) Saturation curves varying 4-oxo-l-proline at the 24 μM NADL level. (B) Saturation curves varying 4-oxo-l-proline at the 12 μM NADL level. (C) Saturation curves varying 4-oxo-l-proline at the 6 μM NADL level. Lines of best fit of the data to eqs 2 and 3are in black and blue, respectively. (D) Saturation curves varying NADL at saturating 4-oxo-l-proline concentration. Lines of best fit of the data to eqs 2 and 4 are in black and blue, respectively. In all cases, L stands for either H or D. All data points from three independent measurements are shown.

**Table 1:** Primary Deuterium Kinetic Isotope Effects on *Hs*BDH2-Catalyzed Reactions^a^

experiment	^D^(*k*_cat_/*K*_M_^Coenzyme^)	^D^(*k*_cat_/*K*_M_^Substrate^)	^D^ *k*_cat_
WT-*Hs*BDH2			
varying 4OLP (24 μM NADL)	N/A	1.6 ± 0.2	1.3 ± 0.1
varying 4OLP (12 μM NADL)	N/A	1.8 ± 0.2	1.4 ± 0.1
varying 4OLP (6 μM NADL)	N/A	1.5 ± 0.3	1.2 ± 0.1
varying NADL (2.0 mM 4OLP)	∼1	N/A	1.3 ± 0.1
varying pyruvate	N/A	1.9 ± 0.2	1.5 ± 0.1
Varying levulinate	N/A	2.6 ± 0.1	2.5 ± 0.1
NADPH as a coenzyme	1.5 ± 0.1	∼1	1.4 ± 0.1
N105-*Hs*BDH2			
pH 7.0	1.7 ± 0.2	2.5 ± 0.2	2.0 ± 0.1
pH 7.5	1.2 ± 0.1	1.6 ± 0.1	2.2 ± 0.2

a Isotope effects are best-fit values ± fitting error from the appropriate equation; N/A stands for not applicable.

The modest and near-unity values of ^D^(*k*_cat_/*K*_M_^4OLP^) and ^D^(*k*_cat_/*K*_M_^NADH^), respectively, suggest the presence of significant commitment factors (C_f_). The C_f_ defines the tendency of a substrate to proceed forward from the Michaelis complex through the isotope-sensitive step up to and including the first irreversible step as opposed to dissociating from the enzyme. For direct-comparison kinetic isotope effects, the C_f_ always refers to the varied substrate, regardless of which substrate carries the isotope label.^21^ The higher the C_f_, the lower the observed kinetic isotope effect on *k*_cat_/*K*_M_, as steps other than chemistry (the only isotope-sensitive step in direct comparison experiments) are rate-limiting.^22,23^ For WT-*Hs*BDH2, dissociation of *cis*-4-hydroxy-l-proline from the enzyme-products ternary complex is presumed to be the first irreversible step (Scheme 2) under initial rate conditions.^10^ For the steady-state ordered mechanism proposed for WT-*Hs*BDH2,^10^ the ^D^(*k*_cat_/*K*_M_^NADH^) could be at least partially unmasked at low concentrations of 4-oxo-l-proline, the second substrate to bind, as the external portion of the C_f_ decreases.^21^ Replotting the data from Figure 2A–C with NADL as varied substrate at 64 μM 4-oxo-l-proline suggests that *K*_M_^NADL^ remain well-below 6 μM (Figure S1B), preventing determination of ^D^(*k*_cat_/*K*_M_^NADH^) even at this very low 4-oxo-l-proline concentration. This raises the possibility that the inherently high affinity of WT-*Hs*BDH2 for NADH (*K*_D_ of 0.48 μM)^10^ is brought about by a slow isomerization of the WT-*Hs*BDH2–NADH binary complex, which would incur an internal C_f_, which is independent of the concentration of the co-substrate.^21^ The effect of isotope-insensitive steps on ^D^ *k*_cat_ is more complex than a C_f_, but such steps will decrease the magnitude of the observed ^D^ *k*_cat_.^21,24^ A relatively high energy barrier to product release is often responsible for a small ^D^ *k*_cat_.^21^ In the case of WT-*Hs*BDH2, this is likely NAD^+^ release to regenerate the free enzyme, as proposed from a pre-steady-state burst of NADH consumption in the forward direction and a very high apparent *K*_M_ for *cis*-4-hydroxy-l-proline in the reverse direction.^10^

### pH-Dependence of ^D^(*k*_cat_/*K*_M_^4OLP^) and ^D^ *k*_cat_

In an attempt to remove the kinetic complexity masking ^D^(*k*_cat_/*K*_M_^4OLP^) and ^D^ *k*_cat_, we determined *k*_cat_/*K*_M_^4OLP^ and *k*_cat_ using NADH and pro-*S* NADD across a pH range (Figure S2) within which catalysis was expected to be affected based on our previous pH-rate profiles.^10^ This is a common strategy used to unveil the expression of kinetic isotope effects as the reaction rate decreases at certain pHs, provided the pH-dependent step includes the isotope-sensitive step (i.e., chemistry).^25^ This has been successfully applied, for instance, to increase the expression of both ^D^(*k*_cat_/*K*_M_) and ^D^ *k*_cat_ at high pH (where *k*_cat_/*K*_M_ and *k*_cat_ decrease) for the *Mycobacterium tuberculosis* dihydrofolate reductase.^14^ The pH-rate profiles with NADH and pro-*S* NADD for WT-*Hs*BDH2 are depicted in Figure 3A, and the variation of ^D^(*k*_cat_/*K*_M_^4OLP^) and ^D^ *k*_cat_ with pH is shown in Figure 3B. Both ^D^(*k*_cat_/*K*_M_^4OLP^) and ^D^ *k*_cat_ remain modest across the pH range tested. Interestingly, ^D^(*k*_cat_/*K*_M_^4OLP^) reduces from a limiting value of 1.77 ± 0.06 at high pH (where *k*_cat_/*K*_M_^4OLP^ is high) to a limiting value of 1.3 ± 0.1 at low pH (where *k*_cat_/*K*_M_^4OLP^ decreases). Meanwhile, ^D^ *k*_cat_ reduces from a limiting value of 1.52 ± 0.02 at low pH (where *k*_cat_ is high) to a limiting value of 1.25 ± 0.02 at high pH (where *k*_cat_ starts to decrease). This points to the isotope-sensitive step differing from the pH-sensitive step in WT-*Hs*BDH2 catalysis at both low and high 4-oxo-l-proline concentrations. The simplest possible explanation for this behavior is that the protonation states of groups relevant for *k*_cat_/*K*_M_^4OLP^ and *k*_cat_ as reflected in the pH-rate profiles are not important for the chemical step, but for other steps in the catalytic cycle. For instance, at low pH, substrate binding, which might include a conformational change of the ternary complex, possibly limits *k*_cat_/*K*_M_^4OLP^, while at high pH, product release, which might comprise a conformational change preceding product diffusion, possibly limits *k*_cat_.

**Figure 3:**
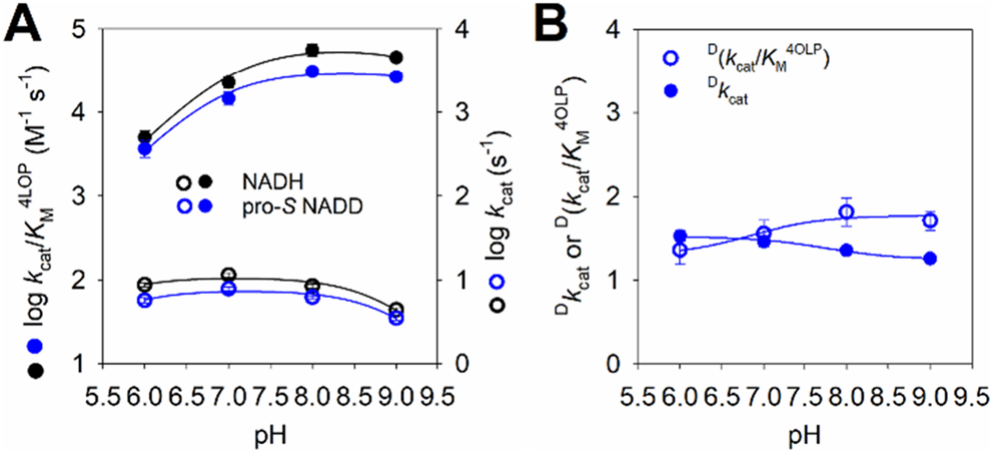
Primary deuterium kinetic isotope effects at different pHs. (A) pH-rate profiles for *k*_cat_ and *k*_cat_/*K*_M_^4OLP^ with NADH and pro-*S* NADD. Data represent the best fit ± fitting error to eq 2 of the data in Figure S2. Lines are the best fit of the data to eq 5. (B) pH-dependence of ^D^ *k*_cat_ and ^D^(*k*_cat_/*K*_M_^4OLP^). The isotope effects were calculated as the ratio of rate constants with NADH to the corresponding rate constants with pro-*S* NADD, and error bars are propagated fitting errors. Lines are the best fit of the data to eq 6.

The solvent viscosity effect previously determined at 9% glycerol at pH 7.0 was unity on *k*_cat_/*K*_M_^4OLP^ and 1.09 on *k*_cat_, indicating that substrate binding was not diffusion-limited, whereas product release had a modest contribution from diffusion.^10^ At pH 8.0, where ^D^(*k*_cat_/*K*_M_^4OLP^) increases and ^D^ *k*_cat_ decreases in relation to their values at pH 7.0, the solvent viscosity effect (Figure S3) is again unity on *k*_cat_/*K*_M_^4OLP^ and 1.17 ± 0.04 on *k*_cat_. This suggests that product diffusion contributes to the rate-limiting step at pH 8.0. The variation of ^D^(*k*_cat_/*K*_M_^4OLP^) with pH is likely the result of non-diffusional steps limiting substrate binding, while the decrease of ^D^ *k*_cat_ at high pH has contributions of diffusional steps limiting product release.

### Solvent Deuterium Kinetic Isotope Effects on WT-*Hs*BDDH2-Catalyzed 4-Oxo-l-proline Reduction by NADH

To probe the contribution of proton-transfer steps to the WT-*Hs*BDH2 reaction rate, we determined pH- and pD-rate profiles from substrate saturation curves in H_2_O and D_2_O (Figure S4), respectively, on *k*_cat_/*K*_M_^4OLP^ and *k*_cat_ (Figure 4). The pH-rate profiles are very similar to those recently reported,^10^ with a bell-shaped *k*_cat_/*K*_M_^4OLP^ profile and a largely pH-independent *k*_cat_. The pD-rate profiles are bell-shaped for both *k*_cat_/*K*_M_^4OLP^ and *k*_cat_. A ^D_2_O^ *k*_cat_ ∼1 is obtained from the pL-independent region (pL 7.0–8.0) of the profile (L stands for either H or D), indicating that proton-transfer steps from the Michaelis complex to the regeneration of the free enzyme are fast in relation to other catalytic steps. The ^D_2_O^(*k*_cat_/*K*_M_^4OLP^) is also ∼1 in the pL-independent region (pL 8.5–9.0) of the profile, indicating that proton transfers occurring from the binding of 4-oxo-l-proline to the enzyme–NADH complex up to and including *cis*-4-hydroxy-l-proline departure are fast relative to other steps. The aforementioned solvent viscosity effects show that the increased viscosity of D_2_O (equivalent to 9% glycerol at 25 °C) does not affect ^D_2_O^(*k*_cat_/*K*_M_^4OLP^) but likely produces an inverse ^D_2_O^ *k*_cat_ of 0.87 ± 0.04 at pH 8.0. The origin of the inverse ^D_2_O^ *k*_cat_ at pH 8.0 is unknown. *Hs*BDH2 catalysis does not involve metal-activated water or cysteine-mediated proton transfer. A possible explanation may be that the ^D_2_O^ *k*_cat_ reflects an inverse ^D_2_O^ *K*_eq_ for a fast proton transfer preceding the rate-limiting product diffusion at pH 8.0.^26^

**Figure 4:**
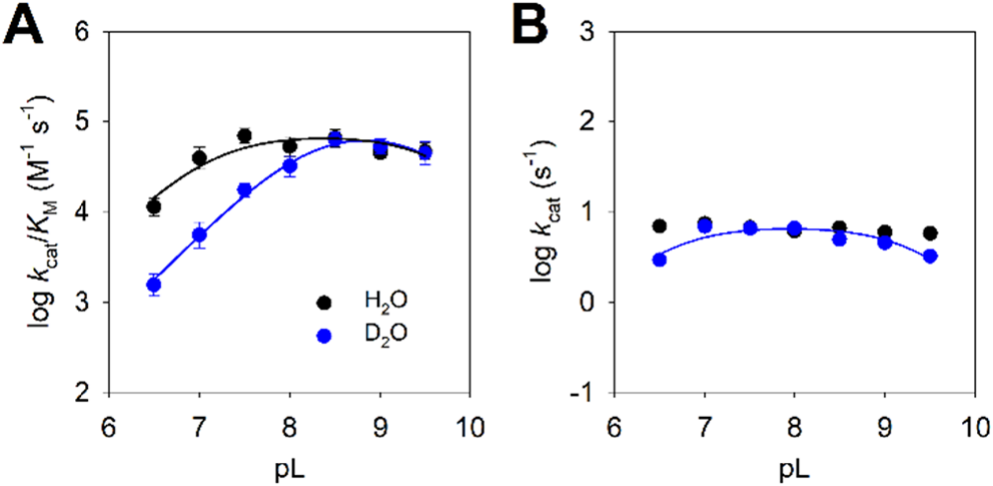
Solvent and multiple deuterium isotope effects. (A) pL-rate profiles for *k*_cat_/*K*_M_^4OLP^ in H_2_O and D_2_O. (B) pL-rate profiles for *k*_cat_ in H_2_O and D_2_O. Data represent the best fit ± fitting error to eq 2 of the data in Figure S4. Lines are the best fit of the data to eq 5. L stands for either H or D. The *k*_cat_ profile in H_2_O was not fitted. All data points are shown for three independent measurements.

The acid-limb apparent p*K*_a_ in the *k*_cat_/*K*_M_^4OLP^ pD-rate profile shifted to 8.1 ± 0.1 from 7.1 ± 0.2 in the pH-rate profile, a higher value than expected from the common increase of ∼0.5 units in the p*K*_a_s of monoprotic acids in D_2_O from their corresponding values in H_2_O.^26^ The origin of this Δp*K*_a_ for *Hs*BDH2 catalysis is not yet known.

### Deuterium Equilibrium Binding Isotope Effects for NADH Binding to WT-*Hs*BDH2

To assess whether the tight binding of NADH to WT-*Hs*BDH2^10^ is accompanied by bond distortion near the reactive center of NADH, i.e., near the nicotinamide C4 position, upon formation of the WT-*Hs*BDH2–NADH binary complex in equilibrium, we determined ^D^ *K*_A_ and ^α‑D^ *K*_A_ (Figure 5). Figure S5 shows representative *m*/*z* data of the NADH standard, used in the deconvolution of the contributions of NADH and NADD to the peaks obtained in the RED experiment. Control experiments lacking the enzyme produced values of 1.02 ± 0.05 for ^D^ *K*_A_ and 1.02 ± 0.01 for ^α‑D^ *K*_A_. It should be pointed out that the nomenclature for primary and α-secondary isotope effects is used here only for ease of identification of which hydrogen is concerned, as in practice, the concept of the primary isotope effect does not apply to equilibrium binding isotope effects because no covalent bond is broken or formed.^16,27^ In the experiment containing the enzyme, an inverse ^D^ *K*_A_ of 0.92 ± 0.01 was obtained, indicating that WT-*Hs*BDH2 binds preferentially to pro-*S* NADD over NADH, and the pro-*S* hydrogen occupies a more constrained bonding environment when NADH is bound to the enzyme instead of free in solution. Conversely, a normal ^α‑D^ *K*_A_ of 1.09 ± 0.01 was measured, showing WT-*Hs*BDH2 binds preferentially to NADH over pro-*R* NADD, and NADH binding to WT-*Hs*BDH2 distorts the pro-*R* hydrogen to a less constrained bonding environment than that in solution.

**Figure 5:**
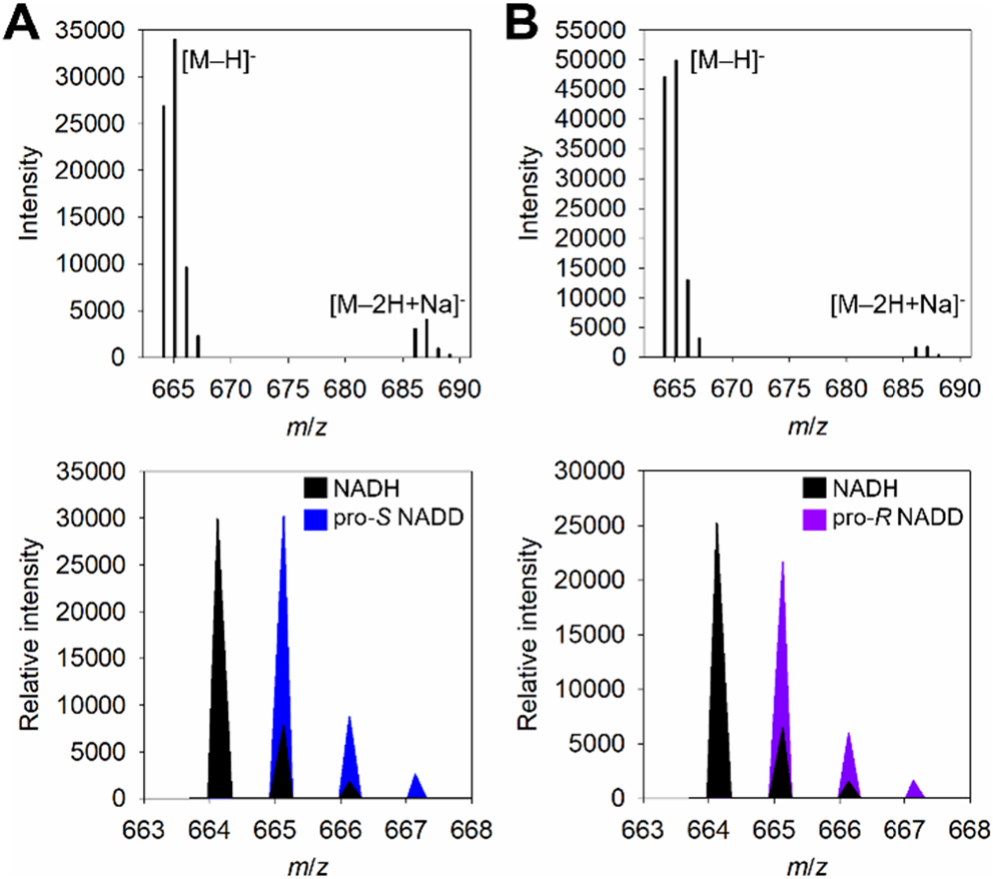
Deuterium equilibrium binding isotope effects on WT-*Hs*BDH2–NADH complex formation. (A) Representative data for ^D^ *K*_A_ measurement. Top: the *m*/*z* detected from an “in” sample, with the mass spectrum of the mixture of NADH and pro-*S* NADD ([M–H]^−^, lower *m*/*z* range) and their Na^+^ adducts ([M–2H+Na]^−^, higher *m*/*z* range). Bottom: representative area plot of the deconvoluted contribution (area under the peaks) from total NADH and pro-*S* NADD (including their Na^+^ adducts). (B) Representative data for ^α‑D^ *K*_A_ measurement. Top: the *m*/*z* detected from an “in” sample, with the mass spectrum of the mixture of NADH and pro-*R* NADD ([M–H]^−^, lower *m*/*z* range) and their Na^+^ adducts ([M–2H+Na]^−^, higher *m*/*z* range). Bottom: representative area plot of the deconvoluted contribution (area under the peaks) from total NADH and pro-*R* NADD (including their Na^+^ adducts).

Isotope effects upon substrate binding are often used to furnish evidence of ground-state destabilization in enzyme catalysis.^28^ In WT-*Hs*BDH2, the normal ^α‑D^ *K*_A_ of 1.09, very similar to the ^α‑D^ *K*_A_ of ∼1.08 reported for the equilibrium binding of NAD^+^ to lactate dehydrogenase,^29^ could represent a sizable portion of the ^α‑D^ *K*_eq_ of 1.12, which might be interpreted as evidence of the enzyme distorting the NADH pro-*R* hydrogen upon binding toward the vibrational environment it occupies in the product NAD^+^, in line with ground-state destabilization with the assumption that it likely occupies a similar vibrational environment at the transition state.

On the other hand, the inverse ^D^ *K*_A_ of 0.92 speaks against ground-state destabilization, since stretching of the C4–H bond in the transition state, as hydride transfer takes place, will produce a normal ^D^ *k*.^30^ Supporting evidence for this should be gleaned from a normal ^D^(*k*_cat_/*K*_M_^NADH^), but NADH tight binding precluded direct measurement of this isotope effect for WT-*Hs*BDH2. It is clear, nonetheless, that the inverse ^D^ *K*_A_ will have dampened the expression of ^D^(*k*_cat_/*K*_M_^NADH^) from the ^D^ *k* even if no C_f_ were present, according to eq 8.^31^

8 (kcatKM)D=KAD×kD

Inverse ^α‑T^ *K*_A_ has been observed for equilibrium binding of uridine to uridine phosphorylase in the absence and presence of sulfate to mimic the co-substrate phosphate,^32^ which contrasts with normal ^α‑T^(*k*_cat_/*K*_M_) and ^α‑T^ *k*.^33^ This was proposed as evidence against ground-state destabilization in uridine phosphorylase catalysis.^32^ With *Hs*BDH2, no unreactive mimic of 4-oxo-l-proline is available to test whether its presence would affect the ^D^ *K*_A_ for NADH and whether evidence for ground-state destabilization could be gleaned from the ^D^ *K*_A_ in the Michaelis complex.

### Primary Deuterium Kinetic Isotope Effects on WT-*Hs*BDDH2-Catalyzed Reduction of Alternative Substrates

To assess WT-*Hs*BDH2’s ability to catalyze the reduction of alternative substrates, we tested the compounds shown in Table S1. Only pyruvate and levulinate produced measurable rates above those of the control lacking enzyme, and their reaction kinetics were further characterized in the presence of a saturating concentration of NADH (Figure S6). With pyruvate, a *k*_cat_ of 0.13 ± 0.01 s^–1^ and a *k*_cat_/*K*_M_ of 26 ± 2 M^–1^ s^–1^ were obtained, reductions of ∼57-fold and 1615-fold in the corresponding rate constants with 4-oxo-l-proline, respectively.^10^ With levulinate, a *k*_cat_ of 0.23 ± 0.01 s^–1^ and a *k*_cat_/*K*_M_ of 6.6 ± 0.6 M^–1^ s^–1^ were obtained, producing reductions of ∼32-fold and 6363-fold in the corresponding rate constants with 4-oxo-l-proline, respectively.^10^

We hypothesized that the WT-*Hs*BDH2-catalyzed slow reductions of pyruvate and levulinate, as compared with that of the physiological substrate, would be the result of slower hydride transfer, which would unmask ^D^(*k*_cat_/*K*_M_) and ^D^ *k*_cat_. We therefore determined kinetic isotope effects for WT-*Hs*BDH2-catalyzed reduction of these low-affinity, slow-reaction substrates (Figure 6). Surprisingly, the ^D^(*k*_cat_/*K*_M_^pyruvate^) was still only 1.9 ± 0.1, and the ^D^ *k*_cat_ was only 1.5 ± 0.1 (Table 1), suggesting that hydride transfer is only moderately rate-limiting with this substrate. For WT-*Hs*BDH2-catalyzed levulinate reduction, however, ^D^(*k*_cat_/*K*_M_^levulinate^) rose to 2.6 ± 0.1 and ^D^ *k*_cat_ to 2.5 ± 0.1 (Table 1). The identical ^D^(*k*_cat_/*K*_M_^levulinate^) and ^D^ *k*_cat_ within experimental error indicate that they are reporting on the same catalytic step, even though *k*_cat_/*K*_M_^levulinate^ reflects steps only up to and including departure of the first product from the WT-*Hs*BDH2–NAD^+^ complex, while *k*_cat_ includes departure of NAD^+^, assuming that the order of substrate binding and product departure is not changed. The latter step is rate-limiting during the reduction of 4-oxo-l-proline,^10^ but it should not have been slowed down or sped up during the reduction of levulinate, since the WT-*Hs*BDH2–NAD^+^ binary complex is the same in both reactions. This points to hydride transfer to levulinate having been substantially slowed down in comparison to 4-oxo-l-proline. Nonetheless, a partially rate-limiting conformational change of the Michaelis complex or of the enzyme-products ternary complex might still be operational, precluding the assertion that ^D^(*k*_cat_/*K*_M_^levulinate^) and ^D^ *k*_cat_ reflect the ^D^ *k* for levulinate reduction. Furthermore, despite both being γ-ketocarboxylates, levulinate and 4-oxo-l-proline differ enough in their structures that the transition states for hydride transfer could be different for these substrates.

**Figure 6:**
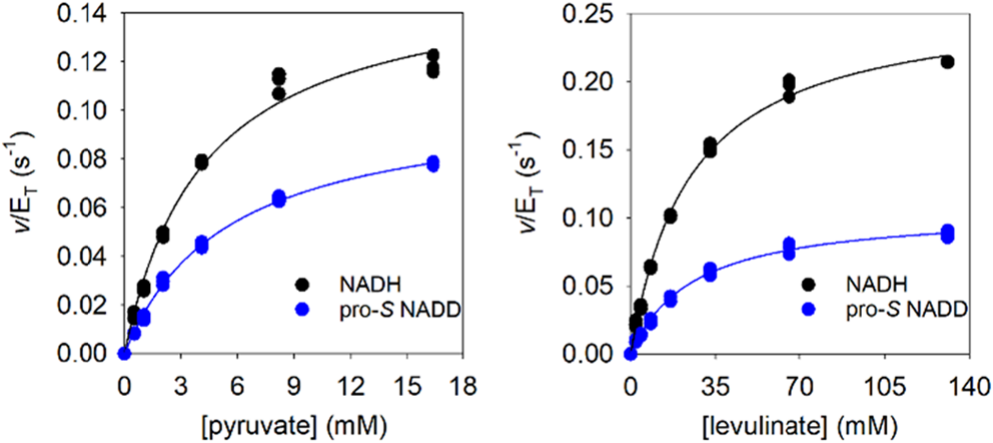
Primary deuterium kinetic isotope effects on WT-*Hs*BDH2-catalyzed reduction of alternative substrates. Saturation curves varying either pyruvate or levulinate at fixed, saturating levels of NADL. Lines of best fit of the data to eqs 2 and 3are in black and blue, respectively. In all cases, L stands for either H or D. All data points from three independent measurements are shown.

### Primary Deuterium Kinetic Isotope Effects on NADPH-Dependent Reduction of 4-Oxo-l-proline

In an attempt to unmask ^D^(*k*_cat_/*K*_M_) and ^D^ *k*_cat_ with a closer mimic to one of the physiological reactants, we assessed the kinetics of WT-*Hs*BDH2-catalyzed reduction of 4-oxo-l-proline with NADPH as the coenzyme. This is a common strategy with NAD­(P)­H-dependent reductases and was successfully employed, for instance, with 1-deoxy-D-xylulose-5-phosphate reductoisomerase^34^ and dihydrofolate reductase,^14^ with the difference that in those cases, NADPH was the physiologically preferred coenzyme, and NADH was used to expose the isotope effects. WT-*Hs*BDH2 steady-state kinetics with NADPH (Figure S7) revealed a *K*_M_^NADPH^ of 170 ± 29 μM, at least a 56-fold increase in comparison with the upper limit for *K*_M_^NADH^ (3 μM).^10^ In contrast, the *K*_M_^4OLP^ of 522 ± 79 μM and the *k*_cat_ of 3.9 ± 0.2 s^–1^ with NADPH represent only a modest increase (∼3-fold) and a modest decrease (∼1.9-fold), respectively, in the corresponding kinetic parameters with NADH.^10^ The measured isotope effects (Figure 7) are summarized in Table 1. The ^D^(*k*_cat_/*K*_M_^NADPH^) increased modestly to 1.5 ± 0.1 from the presumed unity value with NADH, while the ^D^ *k*_cat_ of 1.4 ± 0.1 was unchanged within experimental error. Data of sufficient quality could not be collected at low enough 4-oxo-l-proline concentration with pro-*S* NADPD, such that only an estimate of the ^D^(*k*_cat_/*K*_M_^4OLP^) could be obtained (∼1). The ^D^(*k*_cat_/*K*_M_^NADPH^) above unity and the ^D^(*k*_cat_/*K*_M_^4OLP^) possibly unity suggest that the order of substrate binding to WT-*Hs*BDH2 has been reversed from that with the native coenzyme. Alternatively, given the uncertainty in the ^D^(*k*_cat_/*K*_M_^4OLP^), the kinetic mechanism might have become random instead, provided that the true ^D^(*k*_cat_/*K*_M_^4OLP^) value is slightly above unity.^21^ Since all kinetic isotope effects are still modest, these possible changes in the kinetic mechanism do not alter the conclusion that the use of NADPH did not substantially unmask the primary deuterium kinetic isotope effects for WT-*Hs*BDH2.

**Figure 7:**
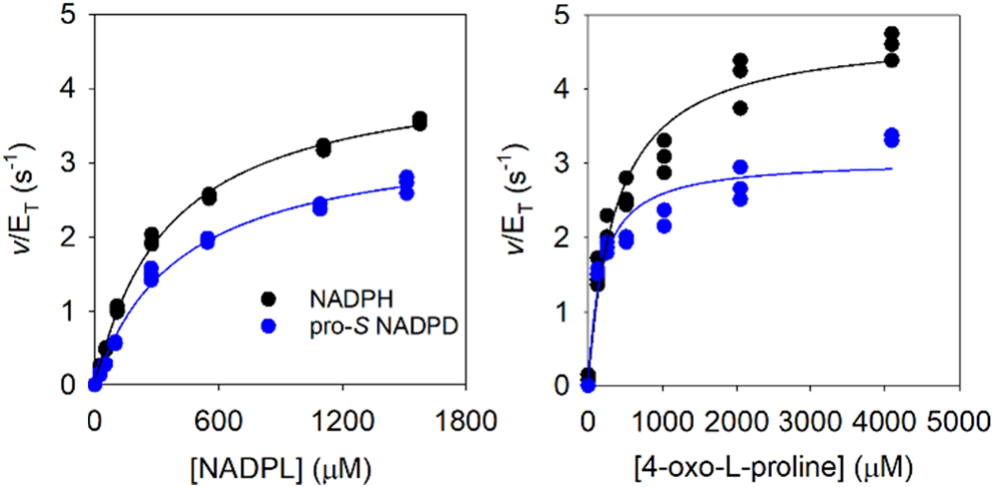
Primary deuterium kinetic isotope effects on NADPH-dependent reduction of 4-oxo-l-proline. Saturation curves for NADPL and 4-oxo-l-proline. Lines of best fit of the data to eqs 2 and 3 are in black and blue, respectively. In all cases, L stands for either H or D. All data points from three independent measurements are shown.

### Effect of N105A Substitution on *Hs*BDH2 Catalysis

To evaluate the importance and possible role in the catalysis of the conserved N105, we produced the N105A-*Hs*BDH2 variant (Figures S8 and S9). The UV–vis absorbance spectrum of the purified N105A-*Hs*BDH2 did not show the peak at 340 nm (Figure S10) seen with WT-*Hs*BDH2,^10^ indicating that this variant does not copurify with NADH. Substrate saturation curves for N105A-*Hs*BDH2 (Figure 8A,B) revealed a *K*_M_^NADH^ of 338 ± 68 μM and a *K*_M_^4OLP^ of 1.5 ± 0.2 mM, ∼113-fold and 9-fold higher, respectively, than the corresponding *K*_M_ (or upper limit thereof) with WT-*Hs*BDH2,^10^ suggesting that the N105A mutation significantly decreases the affinity for the coenzyme and modestly decreases the affinity for the substrate, in qualitative agreement with the proposed role of the conserved N105 in other SDRs.^8^ The *k*_cat_ of 15.3 ± 0.7 s^–1^ is actually double the WT-*Hs*BDH2 *k*_cat_, and it coincidentally matches the single-turnover rate constant.^10^ Therefore, we hypothesized that ^D^(*k*_cat_/*K*_M_) and ^D^ *k*_cat_ would increase for N105A-*Hs*BDH2 in relation to WT-*Hs*BDH2.

**Figure 8:**
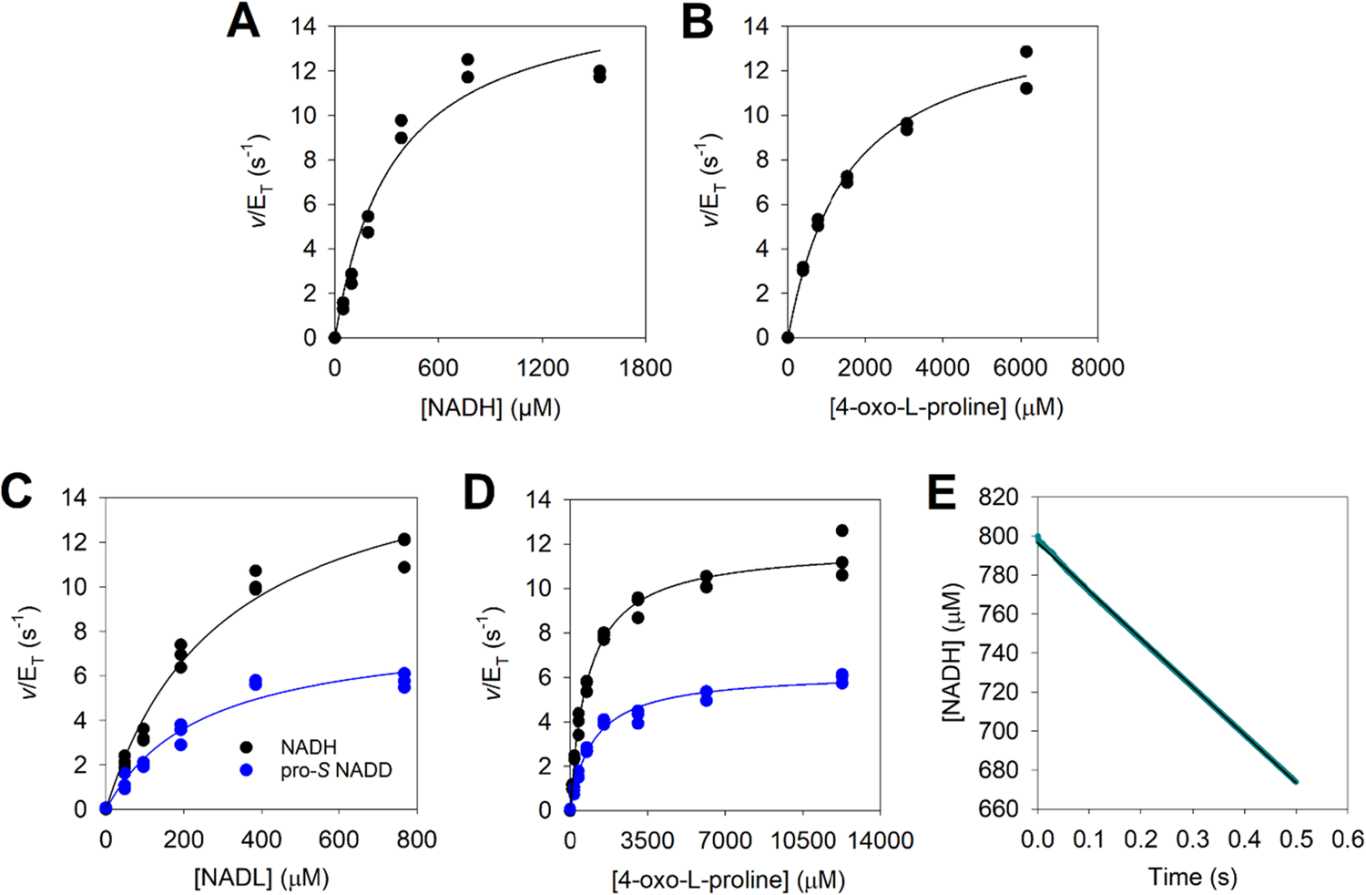
Kinetics with N105A-*Hs*BDH2. (A) Saturation curves for NADH. (B) Saturation curves for 4-oxo-l-proline. Lines of best fit of the data to eq 2 are in black. All data points from two independent measurements are shown. (C) Saturation curves for either NADH or pro-*S* NADD. (D) Saturation curves for 4-oxo-l-proline with either NADH or pro-*S* NADD. Lines of best fit of the data to eqs 2 and 3are in black and blue, respectively. All data points from three independent measurements are shown. L stands for either H or D. (E) Rapid kinetics under multiple-turnover conditions. The line in teal is the averaged data from nine traces. The black line is a linear regression of the data.

The measured isotope effects for N105A-*Hs*BDH2 (Figure 8C,D) are reported in Table 1. As predicted, the N105A substitution reduced the C_f_ for NADH and 4-oxo-l-proline, producing a ^D^(*k*_cat_/*K*_M_^NADH^) of 1.7 ± 0.2 and a ^D^(*k*_cat_/*K*_M_^4OLP^) of 2.5 ± 0.2. This indicates that the kinetic mechanism became random with the N105A mutation.^21^ The ^D^ *k*_cat_ also increased to 2.0 ± 0.1 in comparison with the WT-*Hs*BDH2 ^D^ *k*_cat_ of 1.3. These observations suggest that hydride transfer became significantly rate-limiting for *k*_cat_/*K*_M_^4OLP^ and *k*_cat_ in N105A-*Hs*BDH2 catalysis. This conclusion was further supported by the absence of a burst of NADH consumption in N105A-*Hs*BDH2 rapid kinetics under multiple-turnover conditions (Figure 8E), which produced a steady-state observed rate constant of 16.4 ± 0.1 s^–1^, in agreement with *k*_cat_. The lack of burst kinetics suggests no rate-limiting step following on-enzyme 4-oxo-l-proline reduction by N105A-*Hs*BDH2, in contrast to the burst kinetics observed with WT-*Hs*BDH2, where NAD^+^ release from the enzyme is the likely rate-limiting step.^10^

The role of N105 is therefore proposed to include optimization of hydride transfer in *Hs*BDH2, in addition to the previously proposed impact on coenzyme binding in other SDRs.^8^ This is rationalized by the sizable ^D^(*k*_cat_/*K*_M_^4OLP^) and ^D^ *k*_cat_ for N105A-*Hs*BDH2, the concomitant increase in *k*_cat_, and the disappearance of the pre-steady-state burst kinetics. If coenzyme affinity were the only effect of the mutation, we could expect the increase in ^D^ *k*_cat_ and the lack of burst kinetics due to faster NAD^+^ release from the enzyme, which could also result in a higher *k*_cat_ because of the faster product release rate. However, ^D^(*k*_cat_/*K*_M_^4OLP^) would remain unchanged in comparison with that of WT-*Hs*BDH2, as steps following *cis*-4-hydroxy-l-proline release from the enzyme are not probed by the ^D^(*k*_cat_/*K*_M_^4OLP^). The sizable ^D^(*k*_cat_/*K*_M_^4OLP^) for N105A-*Hs*BDH2 must therefore arise from slower hydride transfer.

### Solvent and Multiple Deuterium Kinetic Isotope Effects on N105A-*Hs*BDDH2-Catalyzed 4-Oxo-l-proline Reduction by NADH

To probe the contribution of proton-transfer steps to the N105A-*Hs*BDH2 reaction rate, we determined pH- and pD-rate profiles on *k*_cat_/*K*_M_^4OLP^ and *k*_cat_ (Figure 9A,B). The pH- and pD-rate profiles are bell-shaped for *k*_cat_/*K*_M_^4OLP^, indicating that a group with a p*K*_a_ of 7.3 ± 0.2 must be deprotonated, and at least one other group must remain protonated for optimal 4-oxo-l-proline binding and/or catalysis; however, the latter group’s p*K*_a_ could not be accurately estimated because not enough data points populate that region of the profile. In the pD profile, the acidic limb p*K*_a_ was displaced to 7.9, whereas the basic limb p*K*_a_ could not be accurately defined, as not enough data points populate that region of the profile. There are no overlapping plateaus between the *k*_cat_/*K*_M_^4OLP^ pH and pD profiles, precluding accurate calculation of the ^D_2_O^(*k*_cat_/*K*_M_^4OLP^) at equivalent pH/pD. The *k*_cat_ profiles are mostly pH/pD-independent, except for the region between pH/pD 6.5 and 7.0. At pH/pD 7.5, the ^D_2_O^ *k*_cat_ in the composite buffer system was 1.4 ± 0.1.

**Figure 9:**
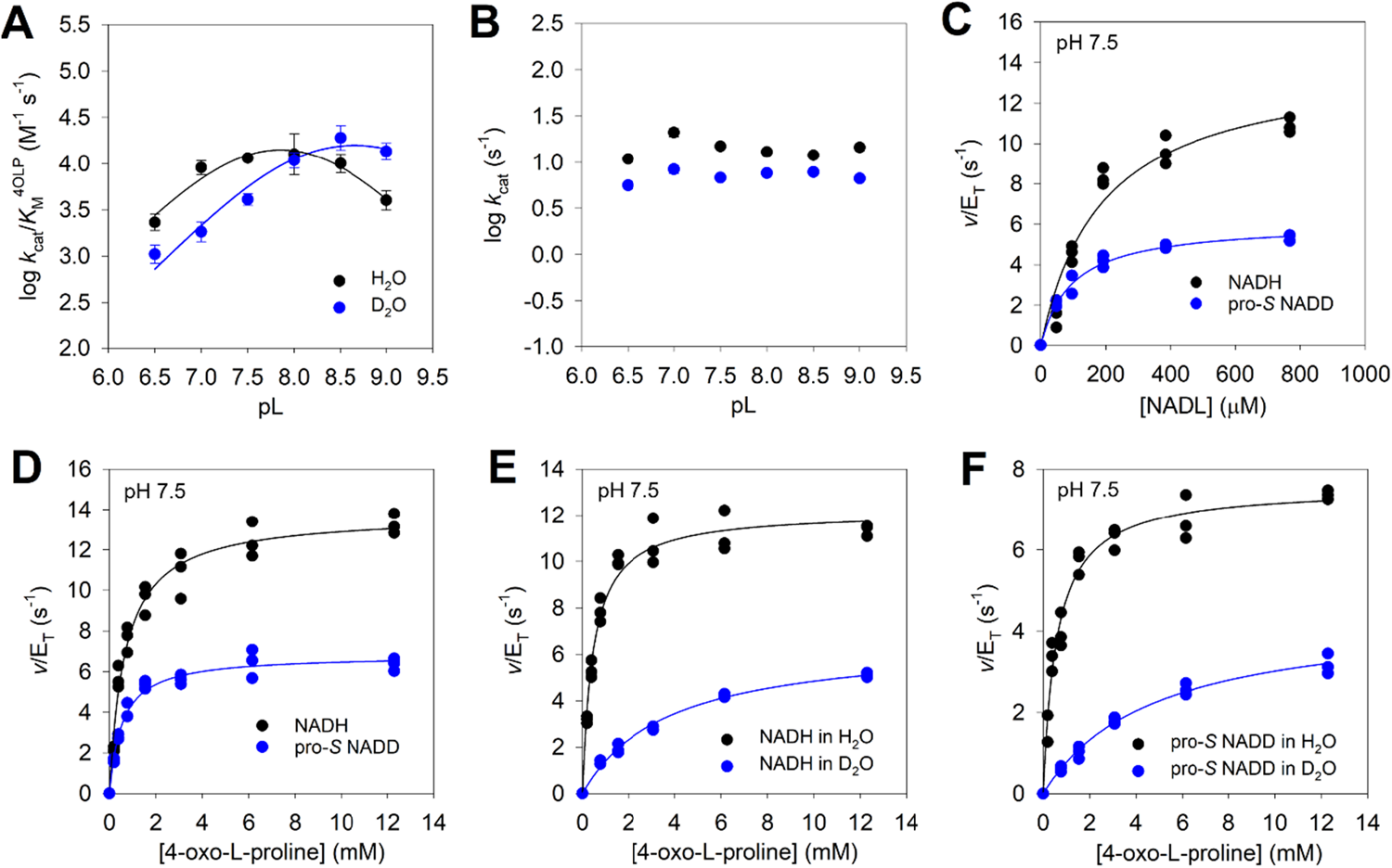
Solvent and multiple deuterium isotope effects for N105A-*Hs*BDH2. (A) pL-rate profiles for *k*_cat_/*K*_M_^4OLP^ in H_2_O and in D_2_O. Lines are the best fit of the data to eq 5. (B) pL-rate profiles for *k*_cat_ in H_2_O and in D_2_O. The *k*_cat_ profile in H_2_O was not fitted. (C) Saturation curves for either NADH or pro-*S* NADD. (D) Saturation curves for 4-oxo-l-proline with either NADH or pro-*S* NADD. (E) Saturation curves for 4-oxo-l-proline at fixed, saturating NADH levels in H_2_O and D_2_O. (F) Saturation curves for 4-oxo-l-proline at fixed, saturating pro-*S* NADD levels in H_2_O and D_2_O. In (C–F), lines of best fit of the data to eqs 2 and 3 are in black and blue, respectively. All data points from three independent measurements are shown. L stands for either H or D.

The ^D^(*k*_cat_/*K*_M_^NADH^) and ^D^(*k*_cat_/*K*_M_^4OLP^) at pH 7.5 decreased to 1.2 ± 0.1 and 1.6 ± 0.1, respectively, from their values at pH 7.0, but the ^D^ *k*_cat_ remained robust at 2.2 ± 0.2 (Figure 9C,D and Table 1). The ^D^ *k*_cat_ at pH 7.5 raises the possibility of using multiple kinetic isotope effects to evaluate the effect on the ^D_2_O^ *k*_cat_ of an increased barrier for hydride transfer when pro-*S* NADD is the coenzyme.^11,35–38^ The ^D_2_O^ *k*_cat_ was 1.9 ± 0.1 when redetermined at pH 7.5 with the same single-buffer system used for ^D^ *k*_cat_ and NADH as the coenzyme (Figure 9E), and the lack of solvent viscosity effects (Figure S3) indicates that the value is not due to increased viscosity with D_2_O. The ^D_2_O^(*k*_cat_)_NADD_, determined under the same conditions except that pro-*S* NADD was the coenzyme, decreased slightly to 1.7 ± 0.1 (Figure 9F). This suggests that the transition state for hydride transfer does not involve a proton transfer, arguing against a concerted mechanism where hydride transfer from NADH and proton transfer from Y247 to 4-oxo-l-proline C4 and O4, respectively, occur in the same transition state in N105A-*Hs*BDH2 catalysis. This mechanism differs from the concerted mechanism reported for related bacterial SDRs catalyzing NADH-dependent reduction of 3-oxovalerate to (*R*)-3-hydroxyvalerate,^11^ but it is in line with a stepwise mechanism proposed for another bacterial SDR catalyzing the NADPH-dependent reduction of β-ketoacyl-ACP to β-hydroxyacyl-ACP,^38^ except that which step takes place first, whether proton transfer or hydride transfer, cannot be ascertained with the current data.

## Conclusions

*Hs*BDH2 catalyzes the NADH-dependent reduction of 4-oxo-l-proline to generate the endogenous anticancer compound *cis*-4-hydroxy-l-proline,^1^ a reaction whose *k*_cat_ is limited by NAD^+^ release from the enzyme.^10^ The inverse ^D^ *K*_eq_ and normal ^α‑D^ *K*_eq_ are in line with the expected bond stiffness of the pro-*S* hydrogen in NADH and *cis*-4-hydroxy-l-proline and the pro-*R* hydrogen in NADH and NAD^+^.^13^ The inverse ^D^ *K*_A_ and normal ^α‑D^ *K*_A_ indicate differential bond distortion experienced by the pro-*S* and pro-*R* hydrogens upon NADH binding to *Hs*BDH2. The modest ^D^(*k*_cat_/*K*_M_^4OLP^) and ^D^ *k*_cat_ point to significant energy barriers for isotope-insensitive steps before and after departure of the *cis*-4-hydroxy-l-proline from the enzyme. This suggests optimized hydride transfer by WT-*Hs*BDH2, resulting in modest ^D^(*k*_cat_/*K*_M_) and ^D^ *k*_cat_, similar to what was recently reported for another human SDR, GDP-L-fucose synthase.^39^ These energy barriers are largely removed during the slow reduction of levulinate, producing sizable ^D^(*k*_cat_/*K*_M_^levulinate^) and ^D^ *k*_cat_, but persist during the slow reduction of pyruvate by NADH and reduction of 4-oxo-l-proline by NADPH. Finally, the sizable ^D^(*k*_cat_/*K*_M_^4OLP^) and ^D^ *k*_cat_ for N105A-*Hs*BDH2 support a direct role for N105 in optimizing hydride transfer, even though the N105A mutation leads to increased *k*_cat_. The decrease of ^D_2_O^(*k*_cat_)_NADD_ from ^D_2_O^(*k*_cat_)_NADH_ suggests stepwise hydride and proton transfers. These results are relevant to other SDRs, where the proposed role of the catalytic tetrad asparagine is limited to coenzyme binding, and exemplify the unique ability of kinetic isotope effects to complement site-directed mutagenesis studies in uncovering the catalytic importance of residues, as it pertains directly to the chemical step.

## Supplementary Material



## Notes

Further experimental results on WT- and N105-*Hs*BDH2, including kinetics, SDS-PAGE, mass spectrometry, and UV–vis spectroscopy (PDF)

## Abbreviations

BDH2: 4-oxo-l-proline reductase
*Hs*BDH2: human BDH2
SDR: short-chain dehydrogenase/reductase
LC-ESI-MS: liquid chromatography-electrospray ionization-mass spectrometry
*K*_D_: equilibrium dissociation constant
*K*_eq_: equilibrium constant
RED: rapid equilibrium dialysis
MES: 2-(N-morpholino)­ethanesulfonic acid
CHES: 2-(cyclohexylamino)­ethanesulfonic acid
HEPES: 4-(2-hydroxyethyl)­piperazine-1-ethanesulfonic acid
^D^(*k*_cat_/*K*_M_): primary deuterium kinetic isotope effect on *k*_cat_/*K*_M_
^D^ *k*_cat_: primary deuterium kinetic isotope effect on *k*_cat_
^D^ *K*_A_: primary deuterium equilibrium isotope effect on *K*_A_
^α‑D^ *K*_A_: α-secondary deuterium equilibrium isotope effect on *K*_A_
^D^ *K*_eq_: primary deuterium equilibrium isotope effect on *K*_eq_
^α‑D^ *K*_eq_: α-secondary deuterium equilibrium isotope effect on *K*_eq_
^D_2_O^(*k*_cat_/*K*_M_^4OLP^): solvent deuterium isotope effect on *k*_cat_/*K*_M_
^D_2_O^ *k*_cat_: solvent deuterium isotope effect on *k*_cat_
