# Supplementary material for: Probing the Human 4‑Oxo‑l‑proline Reductase-Catalyzed Reaction by Deuterium Kinetic and Equilibrium Isotope Effects
Source: Biochemistry. 2026 Jul 16;65(15):2419–30. doi: 10.1021/acs.biochem.6c00491 (PMC13445582; doi:10.1021/acs.biochem.6c00491)
Supplement: Supplementary file 1 [file bi6c00491_si_001.pdf]

Supporting Information

Probing the human 4-oxo-L-proline reductase-catalysed reaction by deuterium kinetic and equilibrium isotope effects

Ennio Pečaver[†,⊥], Myriam Kabu[†,⊥], Greice M. Zickuhr[§], David J. Harrison[§,£], Rafael G. da Silva[†,*]

[†]School of Biology, Biomedical Sciences Research Complex, University of St Andrews, St Andrews, KY16 9ST, United Kingdom

[§]School of Medicine, University of St Andrews, St Andrews, KY16 9TF, United Kingdom

[£]NuCana Plc, Edinburgh EH12 9DT, United Kingdom

[⊥]These authors contributed equally to the work

*To whom correspondence may be addressed: rgds@st-andrews.ac.uk, phone: +44 01334 463496

Table S1. Compounds tested as alternative substrate for *Hs*BDH2.

Compound	Structure
4-Nitrobenzaldehyde	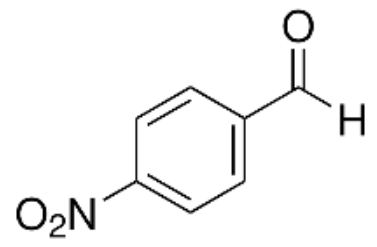
DL-Glyceraldehyde	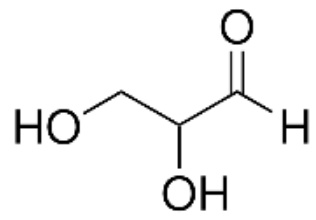
L-Pyroglutamic acid	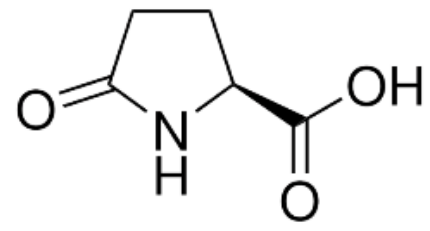
(R)-(-)-5-oxo-2-Tetrahydrofuran Carboxyl	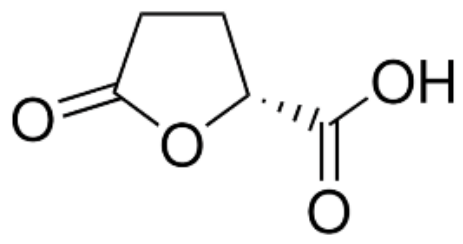
Clavulanate	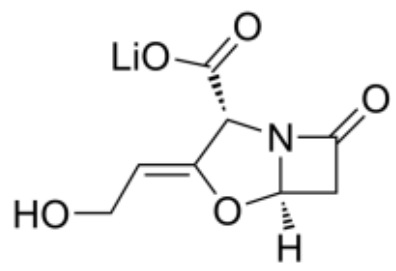
2-Azetidinone	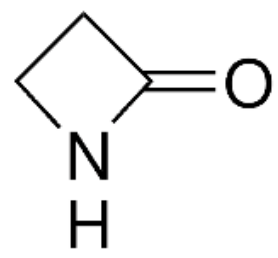
2-Methyl-2-Cyclopenten-1-One	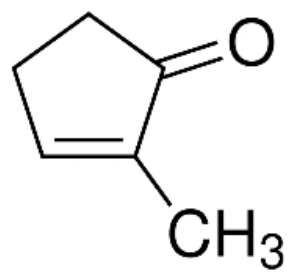
4-Methyl-2-oxovaleric acid	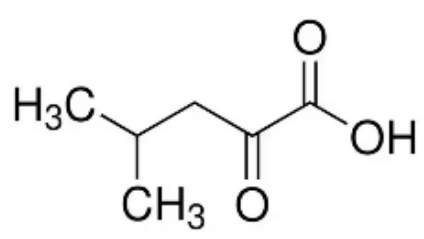
4-Methyl-3-Penten-2-one	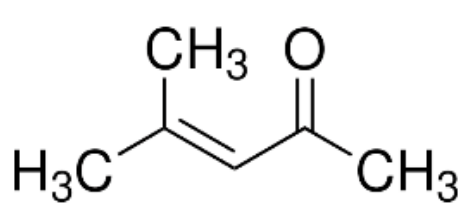

α-Ketoglutaric acid	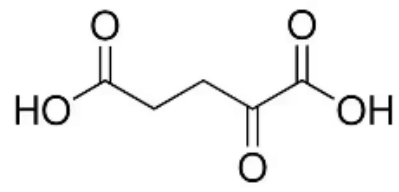
5-AminoLevulinic Acid	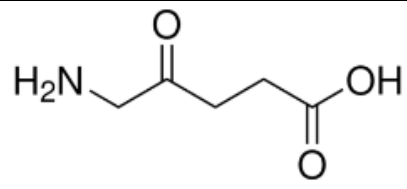
Levulinic Acid	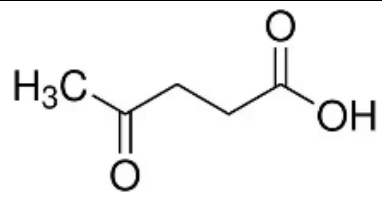
Pyruvic acid	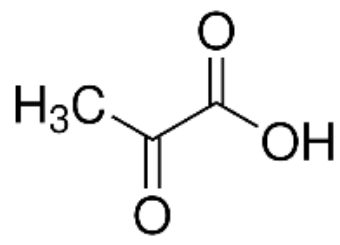
2-Ketobutyric acid	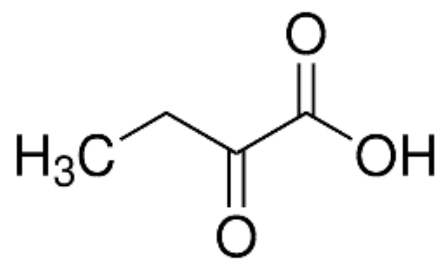
Metformin	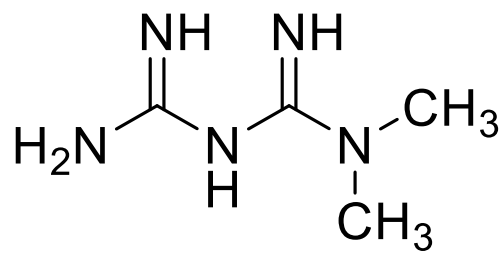

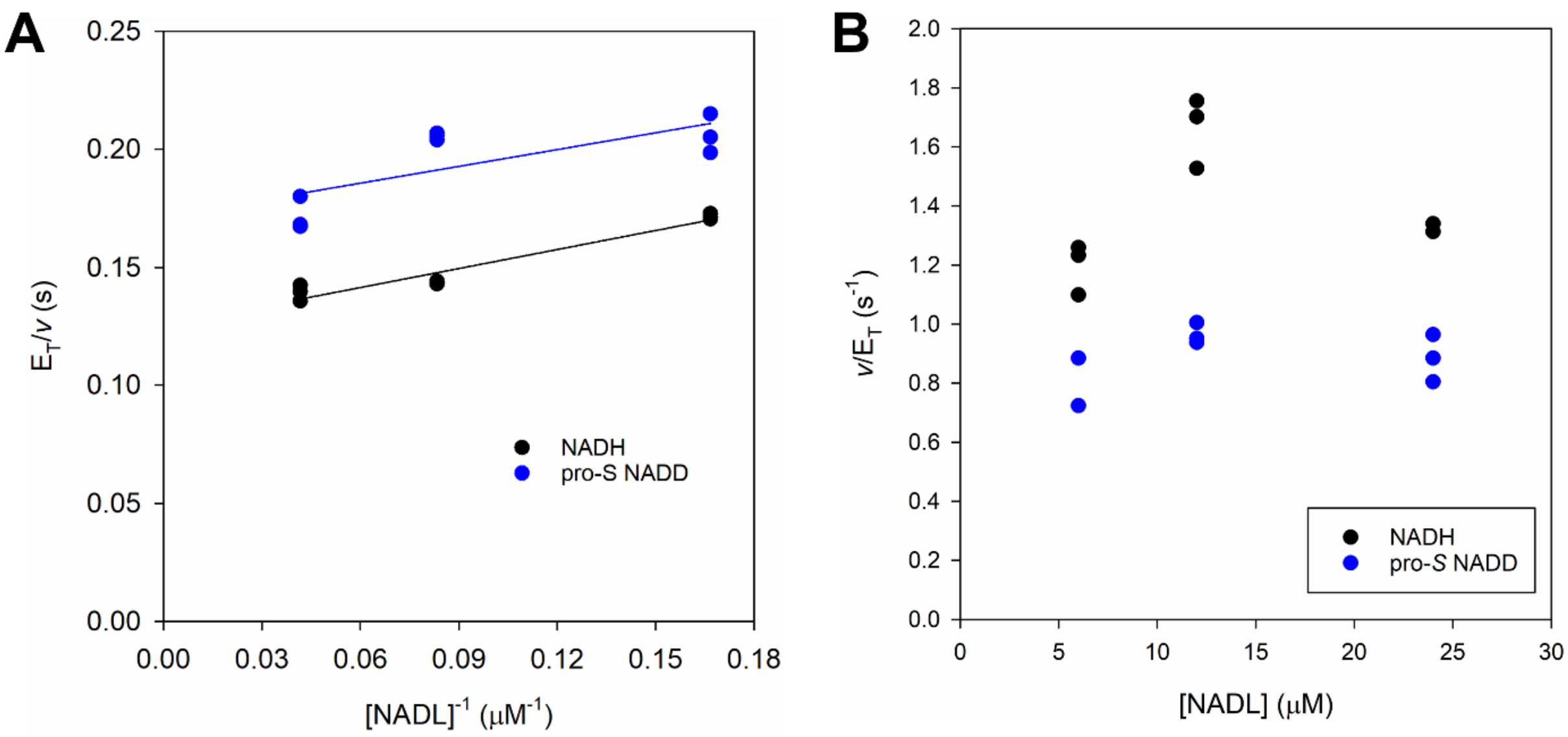

Figure S1. (A) Double-reciprocal plot of WT-*Hs*BDH2 saturation curves at varying concentrations of either NADH or pro-*S* NADD, and a fixed, saturating concentration of 4-oxo-L-proline. Lines are linear regressions of the data. **(B)** NADL saturation curves at 64 μM 4-oxo-L-proline, a concentration well-below its K_M. All data points are shown from three independent measurements. L stands for either H or D.

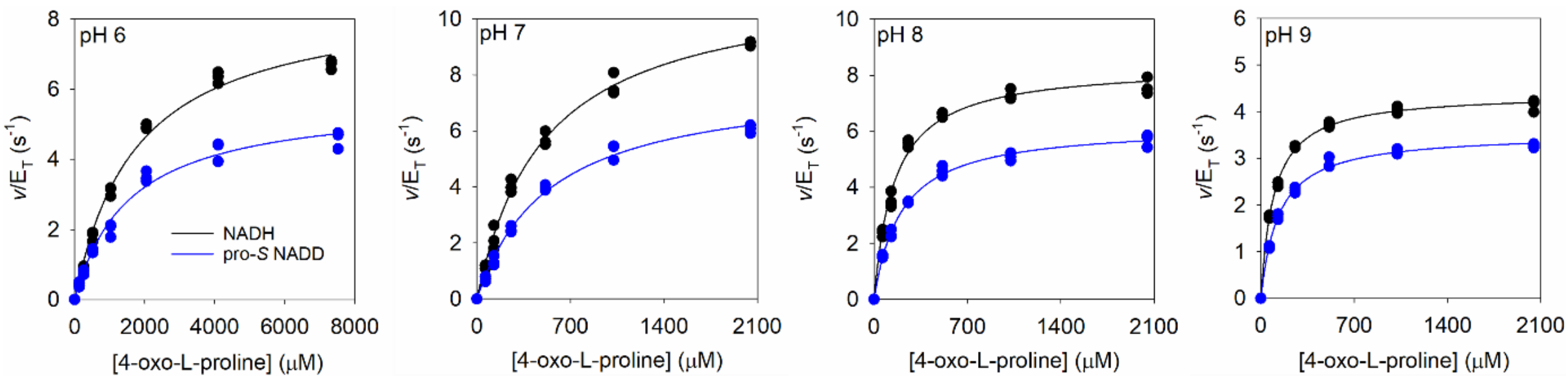

Figure S2. WT-*Hs*BDH2 saturation curves for 4-oxo-L-proline at fixed, saturating concentrations of either NADH or pro-*S* NADD at different pHs. Lines are best fit of the data to eq 2. All data points are shown from three independent measurements.

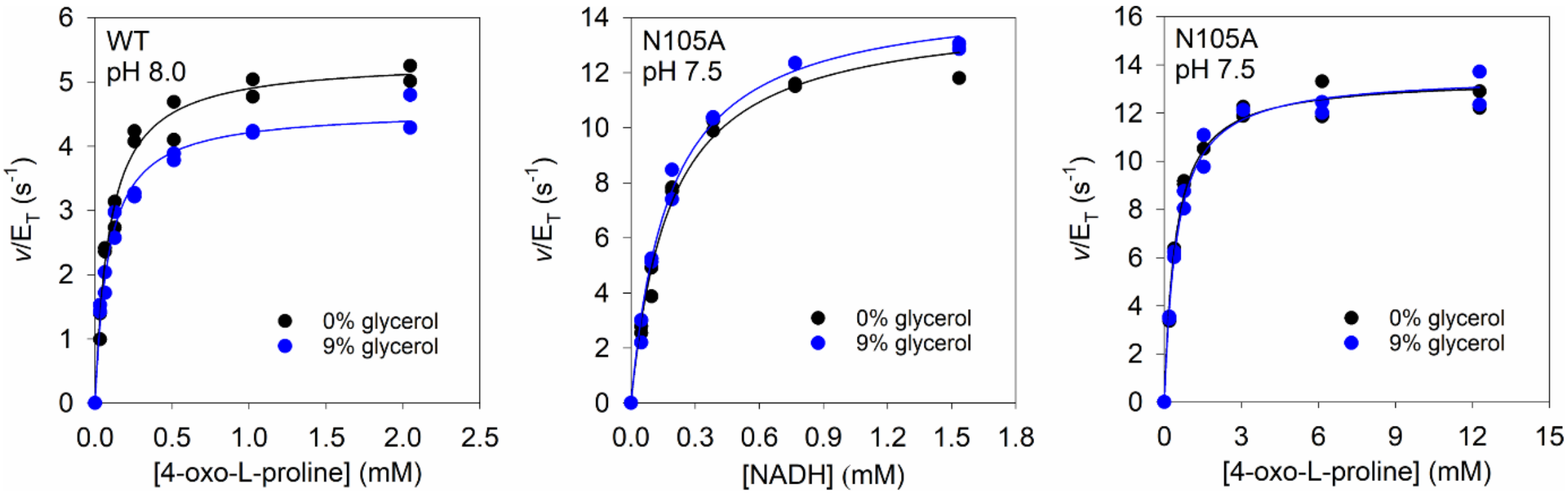

Figure S3. Substrate saturation curves in the presence and absence of 9% glycerol (v/v). All data points are shown from two independent measurements. Lines are best fit of the data to eq 2.

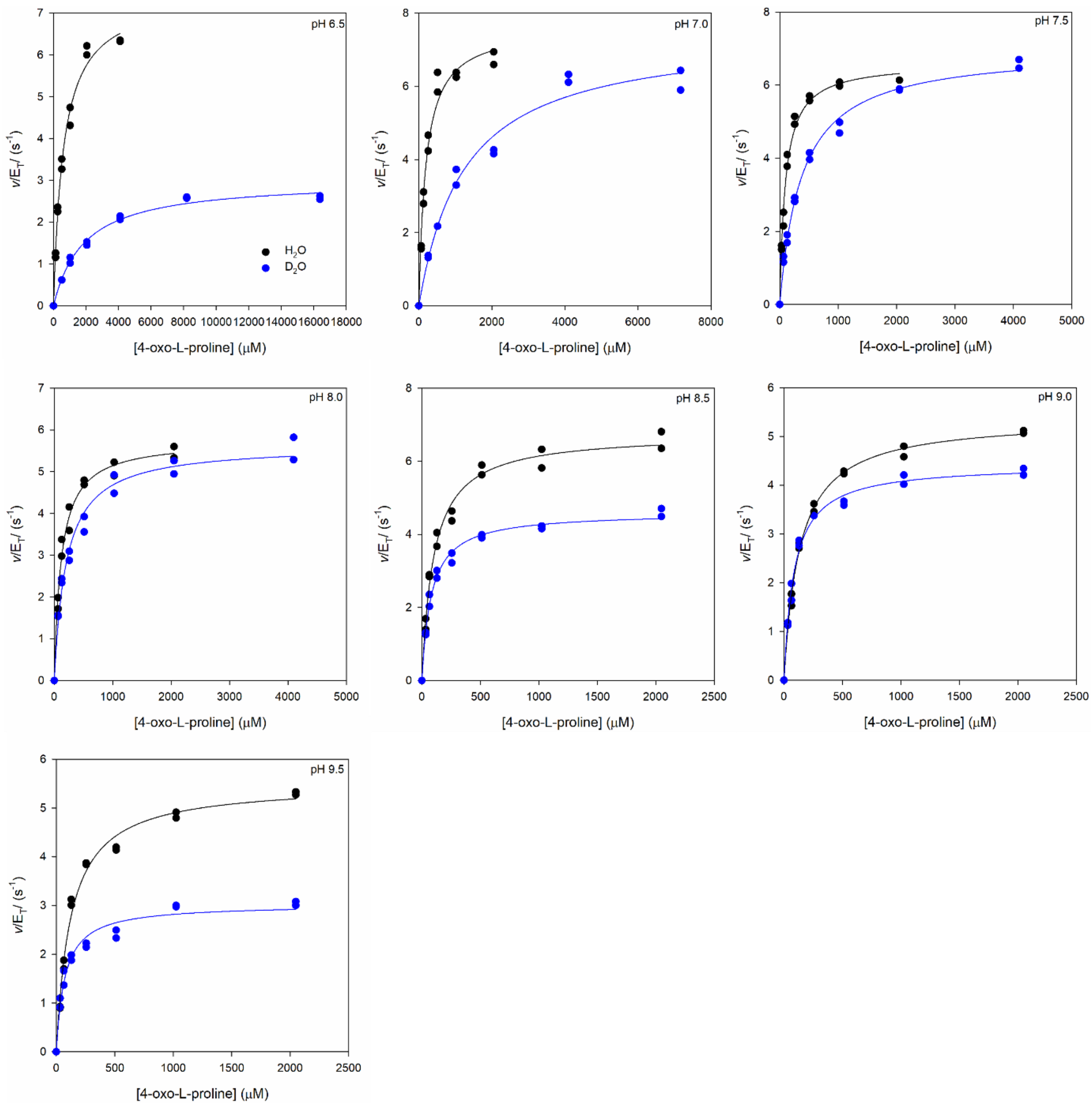

Figure S4. WT-*Hs*BDH2 saturation curves for 4-oxo-L-proline at fixed, saturating concentrations of NADH at different pHs (or pDs). Lines are best fit of the data to eq 2. All data points are shown from two independent measurements.

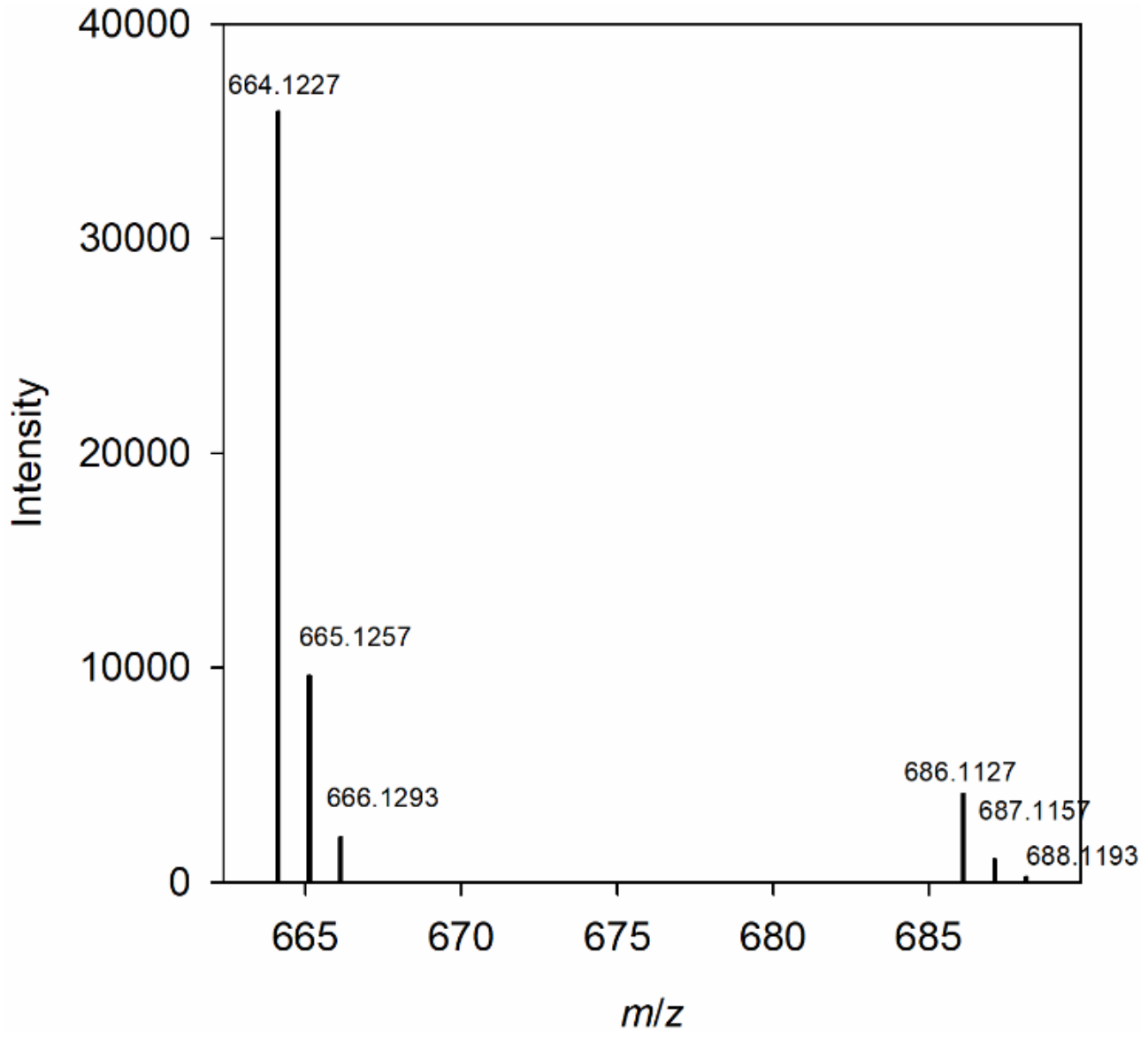

Figure S5. HRMS spectrum of the NADH standard, including the Na^{+}-adduct peaks at higher *m/z* range.

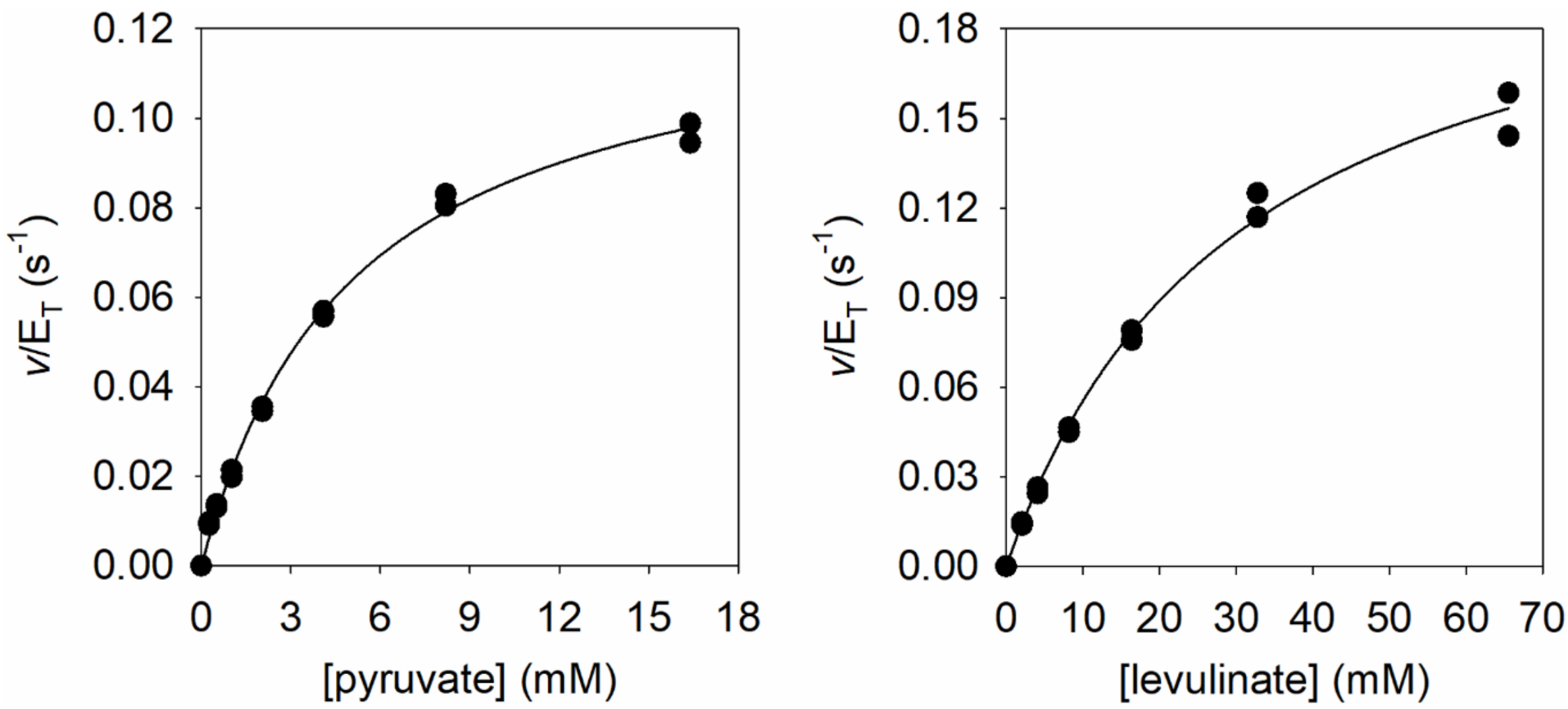

Figure S6. WT-*Hs*BDH2 saturation curves for pyruvate and levulinate at fixed, saturating concentrations of NADH. Lines are best fit of the data to eq 2. All data points are shown from two independent measurements.

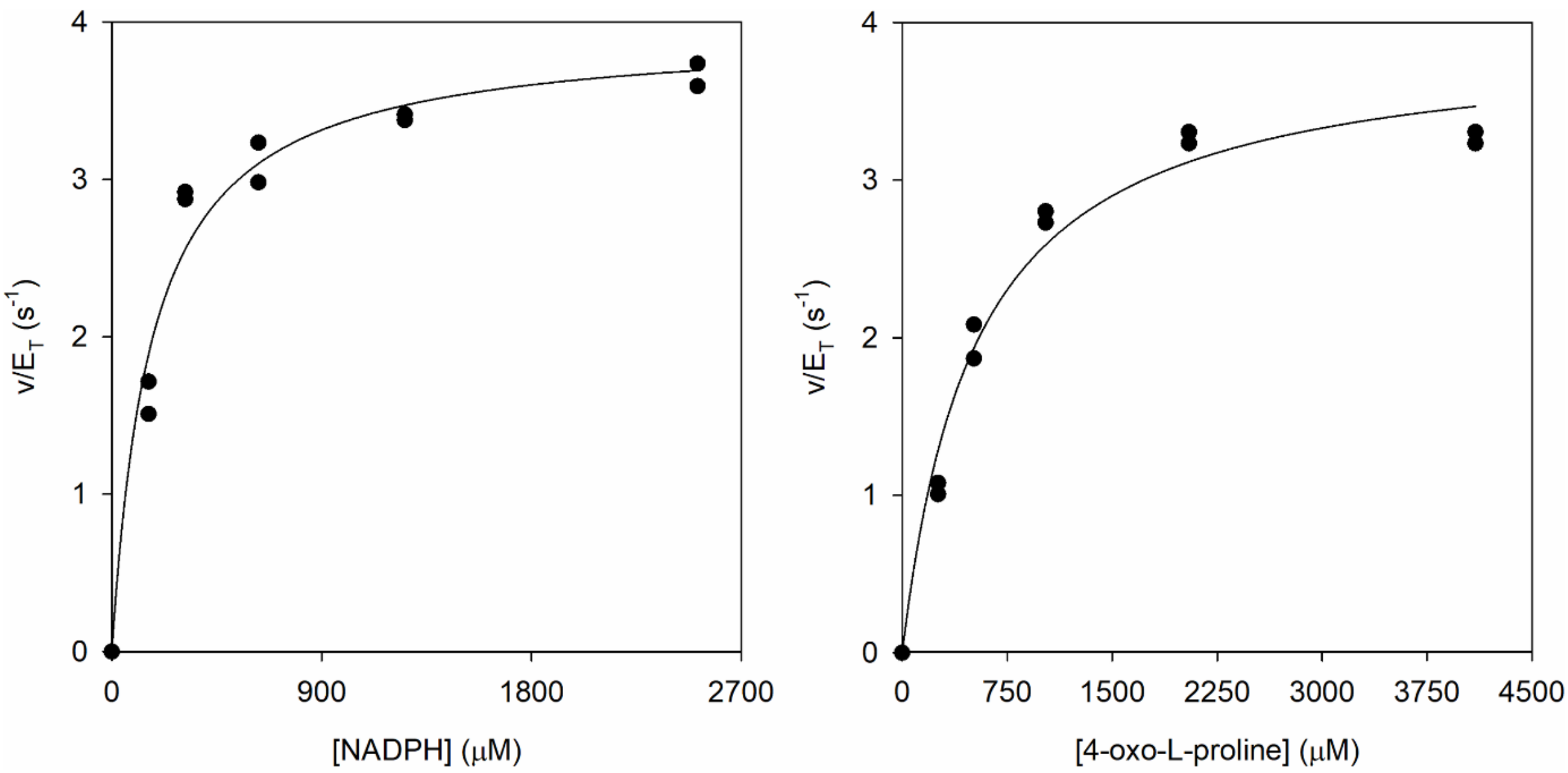

Figure S7. WT-*Hs*BDH2 saturation curves with NADPH as coenzyme. In each case, the fixed substrate concentration was saturating. Lines are best fit of the data to eq 2. All data points are shown from two independent measurements.

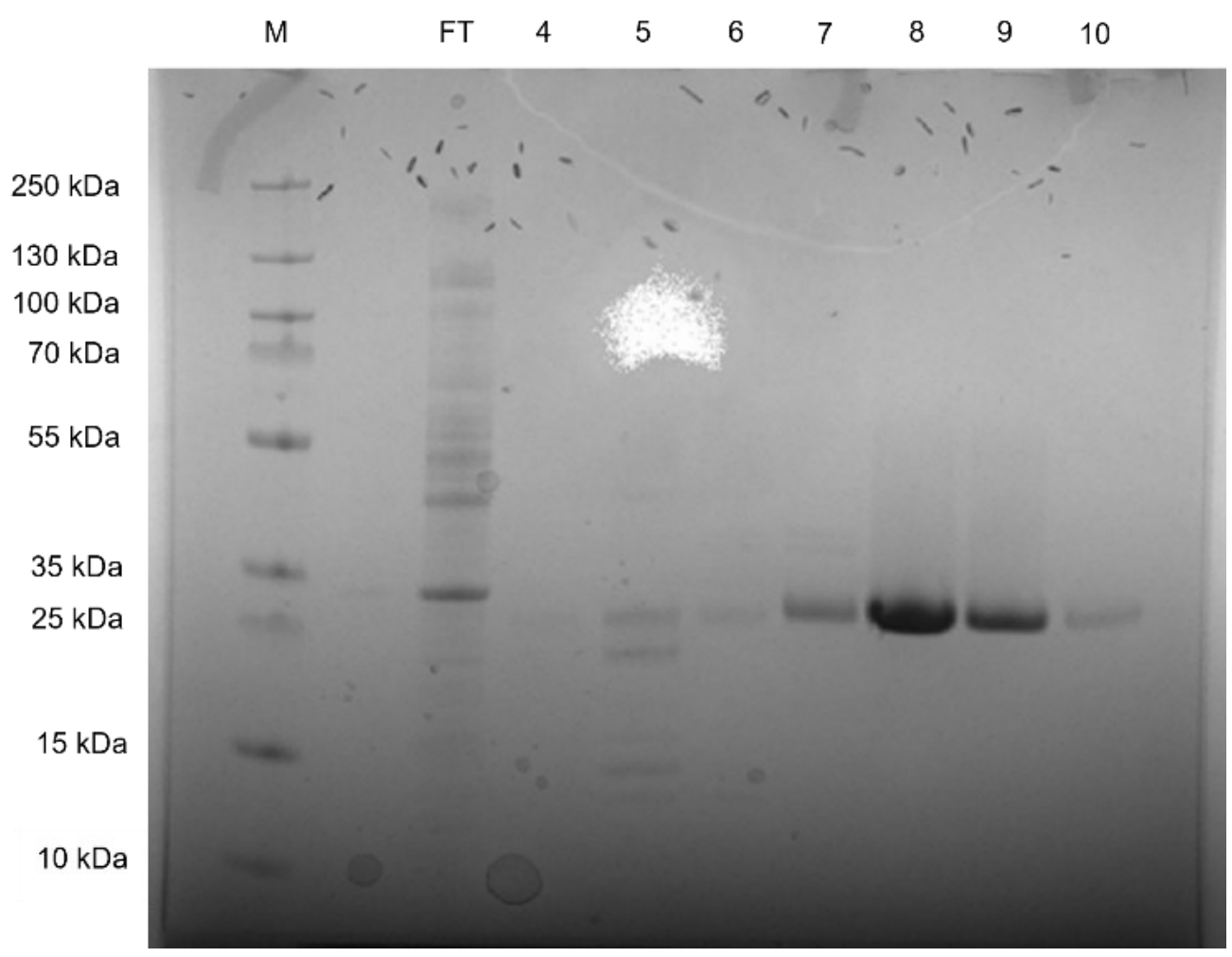

Figure S8. SDS-PAGE of N105A-*Hs*BDH2 fractions eluted from the HisTrap FF column. Lane M is the PageRuler Plus Prestained MWM, lane FT is the flowthrough, and lanes 4 – 10 are eluted fractions. Fractions 8 – 10 were pooled.

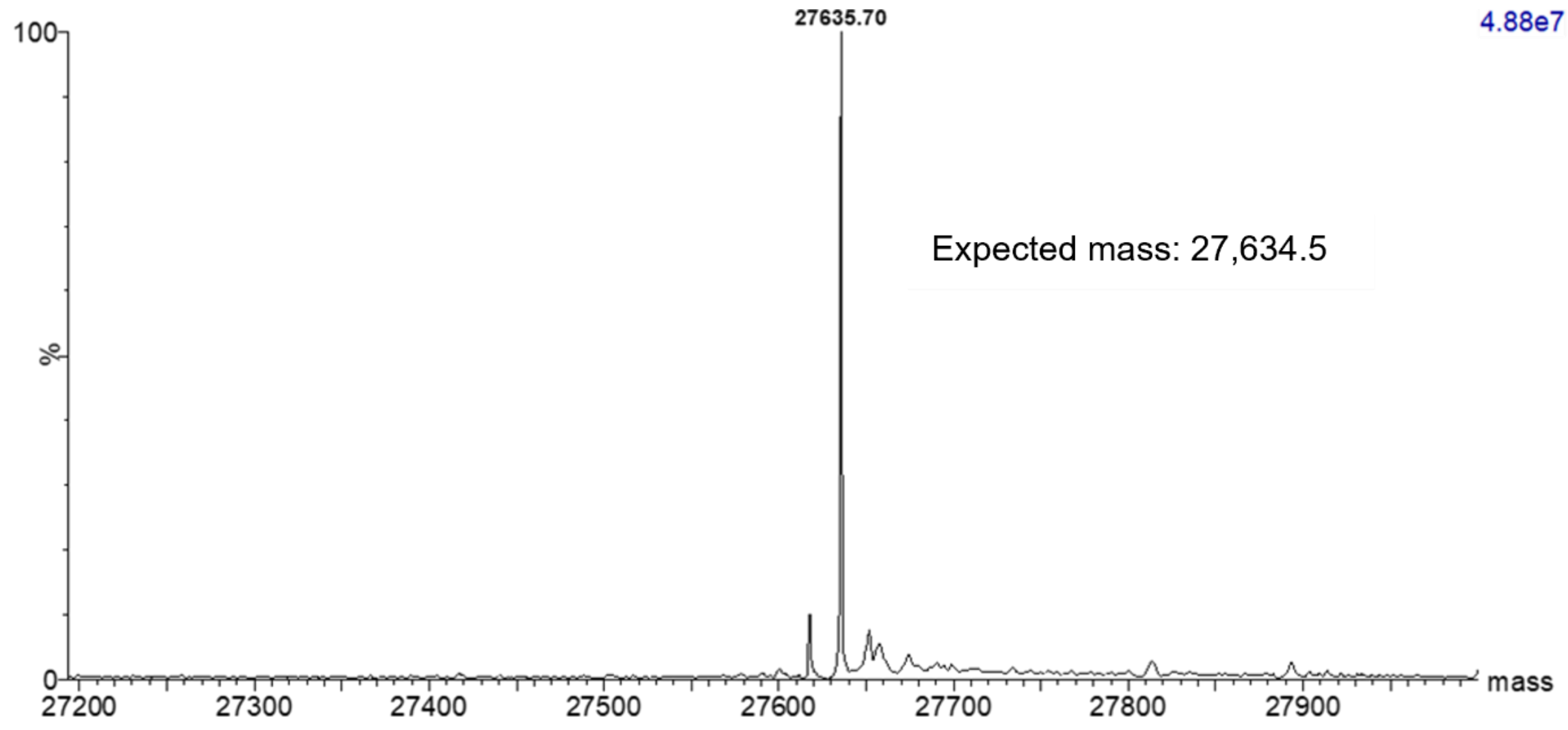

Figure S9. LC-ESI-MS spectrum of purified N105A-*Hs*BDH2.

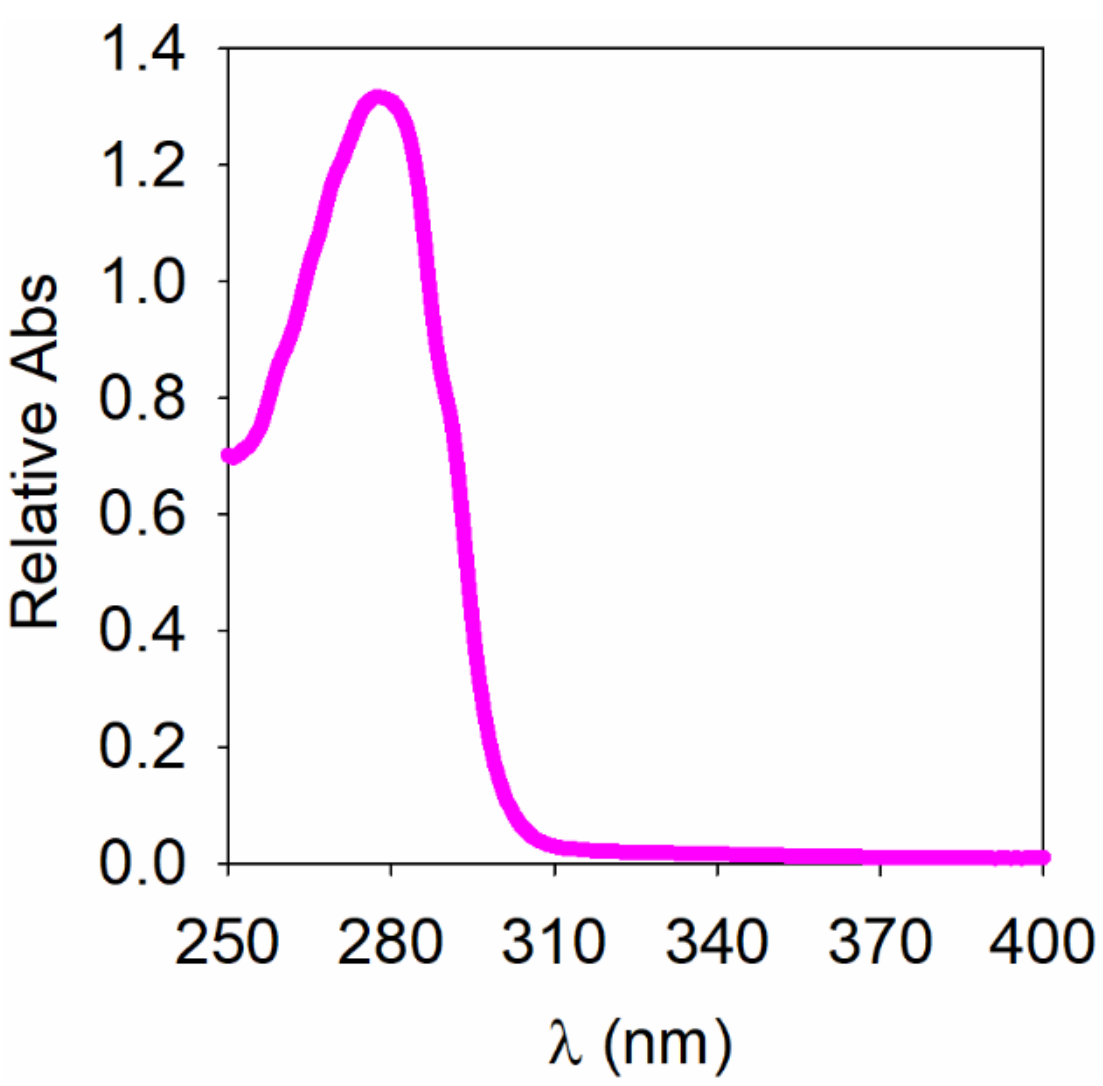

Figure S10. UV-VIS spectrum of purified N105A-*Hs*BDH2. The protein concentration was 69 μM in assay buffer, the cuvette pathlength was 1 cm. The spectrophotometer was blanked with the assay buffer.
